# Human APOBEC3 Induced Mutation of Human Immunodeficiency Virus Type-1 Contributes to Adaptation and Evolution in Natural Infection

**DOI:** 10.1371/journal.ppat.1004281

**Published:** 2014-07-31

**Authors:** Eun-Young Kim, Ramon Lorenzo-Redondo, Susan J. Little, Yoon-Seok Chung, Prabhjeet K. Phalora, Irina Maljkovic Berry, John Archer, Sudhir Penugonda, Will Fischer, Douglas D. Richman, Tanmoy Bhattacharya, Michael H. Malim, Steven M. Wolinsky

**Affiliations:** 1 Division of Infectious Diseases, Northwestern University Feinberg School of Medicine, Chicago, Illinois, United States of America; 2 Division of Infectious Diseases, University of California San Diego, San Diego, California, United States of America; 3 Department of Infectious Diseases, King's College London, Guy's Hospital, London, United Kingdom; 4 Faculty of Life Sciences, University of Manchester, Manchester, United Kingdom; 5 Los Alamos National Laboratory, Los Alamos, New Mexico, United States of America; 6 Veterans Affairs San Diego Healthcare System, San Diego, California, United States of America; 7 Santa Fe Institute, Santa Fe, New Mexico, United States of America; University of Massachusetts Medical School, United States of America

## Abstract

Human APOBEC3 proteins are cytidine deaminases that contribute broadly to innate immunity through the control of exogenous retrovirus replication and endogenous retroelement retrotransposition. As an intrinsic antiretroviral defense mechanism, APOBEC3 proteins induce extensive guanosine-to-adenosine (G-to-A) mutagenesis and inhibit synthesis of nascent human immunodeficiency virus-type 1 (HIV-1) cDNA. Human APOBEC3 proteins have additionally been proposed to induce infrequent, potentially non-lethal G-to-A mutations that make subtle contributions to sequence diversification of the viral genome and adaptation though acquisition of beneficial mutations. Using single-cycle HIV-1 infections in culture and highly parallel DNA sequencing, we defined trinucleotide contexts of the edited sites for APOBEC3D, APOBEC3F, APOBEC3G, and APOBEC3H. We then compared these APOBEC3 editing contexts with the patterns of G-to-A mutations in HIV-1 DNA in cells obtained sequentially from ten patients with primary HIV-1 infection. Viral substitutions were highest in the preferred trinucleotide contexts of the edited sites for the APOBEC3 deaminases. Consistent with the effects of immune selection, amino acid changes accumulated at the APOBEC3 editing contexts located within human leukocyte antigen (HLA)-appropriate epitopes that are known or predicted to enable peptide binding. Thus, APOBEC3 activity may induce mutations that influence the genetic diversity and adaptation of the HIV-1 population in natural infection.

## Author Summary

Cytidine deaminases of the human APOBEC3 gene family act as an intrinsic defense mechanism against infection with HIV-1 and other viruses. The APOBEC3 proteins introduce mutations into the viral genome by inducing enzymatic modification of nucleotide sequences and inhibiting synthesis of cDNA strands from the viral RNA. Viral Vif counters this impediment to the fidelity of HIV-1 replication by targeting the APOBEC3 proteins for degradation. Low-level APOBEC3 activity that outlasts blockade by viral Vif may foster infrequent mutations that provide a source of genetic variation upon which natural selection acts. Here, we defined the APOBEC3 nucleotide contexts of the edited sites by titration of the wild type and non-editing APOBEC3 mutant in cultured cells. We then followed the patterns of G-to-A mutations we identified in viral DNA in cells obtained from ten patients with acute infection. Our deep sequencing analyses demonstrate an association between sub-lethal APOBEC3 editing and HIV-1 diversification. Mutations at APOBEC3 editing contexts that occurred at particular positions within specific known or predicted epitopes could disrupt peptide binding critical for immune control. Our findings reveal a role for human APOBEC3 in HIV-1 sequence diversification that may influence fitness and evolution of beneficial variants and phenotypes in the population.

## Introduction

The pathogenesis of HIV-1 infection correlates with the level of active viral replication and relates to a variety of factors specific to the virus, the host, and its immune system. Mutations, insertions, deletions and recombinations that confer changes in the activity of virally encoded genes and gene products affect virus entry and post-entry events [1]. HIV-1 shows high mutational frequency because of a combination of rapid rates of viral replication, error-prone viral reverse transcriptase (RT) and RNA polymerase II replicating enzymes, and recombination during concurrent infection with two or more distinct genomic RNA strands [2]–[4]. In addition, nascent HIV-1 cDNA is vulnerable to mutation by host cell single-stranded cytidine deaminases that edit cytidine to uridine in the minus strand DNA copied from the viral RNA genome, giving rise to G-to-A mutation of the plus strand of viral DNA with a graded frequency of deamination from the primer binding site to the central polypurine tract and the central polypurine tract to the 3′ polypurine tract regions [5]–[8]. The effect of the processes of mutation and recombination in HIV-1 is to promote genetic diversity among the viral variants and thereby allow for a faster rate of adaptation.

Seven related human apolipoprotein B mRNA-editing enzyme, catalytic polypeptide-like 3 (APOBEC3) cytidine deaminases—namely, *APOBEC3A*, *APOBEC3B*, *APOBEC3C*, *APOBEC3D*, *APOBEC3F*, *APOBEC3G*, and *APOBEC3H*—reside in an expanded 130-kb gene cluster that likely arose through segmental duplication on chromosome 22q13.1 with additional modification by repeated episodes of positive selection during primate evolution [9]. Cytidine deaminases of the APOBEC3 gene family have specificity for single-stranded DNA and inhibit infection by a diverse array of RNA and DNA viruses and retrotransposons by interfering with viral genome replication and littering the genome with deleterious mutations [10], [11]. Mutations mediated by APOBEC3 molecules have a strong preference for a 5′-GG-3′ and 5′-GA-3′ dinucleotide context of the edited sites (target nucleotide underlined) [1], [12], [13].

APOBEC3D, APOBEC3F, APOBEC3G and APOBEC3H are the cellular targets for the HIV-1 accessory protein Vif [14], [15], which can counteract the protective role of this innate immune defense mechanism. The HIV-1 protein Vif induces polyubiquitylation through simultaneously binding to APOBEC3 proteins and the cullin5-elongin B/C-Rbx2 ubiquitin ligase complex. In this manner, the APOBEC3 protein serves as an adaptor that recruits the ligase complex to its substrate and induces the subsequent proteasomal degradation of APOBEC3 proteins. This depletes the pool of APOBEC3 proteins available for incorporation into the assembling viral particle, and thereby minimizes their ability to restrict HIV-1 replication [16]–[19].

Mutation of the HIV-1 genome by cytidine deamination could have a dramatic effect on viral replication. A high rate of mutation could prevent the formation of functional proviruses and explain the G-to-A hypermutation observed in patient samples [20], [21]. Low levels of APOBEC3 activity that survive inhibition by the HIV-1 protein Vif may expose the virus to a broad spectrum of mutations that, rather than impeding virus replication, could provide a source of genetic variation upon which natural selection acts [22], [23]. Indeed, extensive sequencing of transmitted founder HIV-1 variants indicates that sequence variation bearing the hallmark of APOBEC3-mediated G-to-A mutation is commonplace and experiments employing low levels of APOBEC3G expression confirm that modest mutation frequencies, as opposed to inactivating G-to-A hypermutation (5′-UGG-3′ to 5′-UAG-3′; tryptophan-to-stop codon), can be recapitulated in cell culture experiments [21], [24]–[26]. Such sequence changes would have the potential to underlie advantageous alterations in HIV-1 phenotype, such as the appearance of mutations in HLA class I-restricted epitopes that can confer escape from immune recognition or the acquisition of drug resistance [1], [21], [22], [27].

Here, we characterized the relationship(s) between APOBEC3 editing of HIV-1 and the perhaps subtle contribution of mutations that could influence viral adaptation and evolution, as contrasted with destructive G-to-A hypermutation. We applied high depth sequencing to infected cells from single-cycle APOBEC3 titration transfection experiments to confirm and extend the definitions of the DNA sequence context of the edited sites for the four cytidine deaminases of the APOBEC3 gene family that are the cellular targets for HIV-1 protein Vif and their site-specific editing frequencies [5], [7], [8], [12], [13], [28]–[30]. We then followed the evolution of sequence changes and the appearance of the G-to-A signature mutations in the consensus trinucleotide contexts for APOBEC3 protein edited sites in the Gag and Vif genes of HIV-1 in proviral DNA sampled from the peripheral blood in 10 patients with primary HIV-1 infection through time. We found a higher frequency of substitutions within an APOBEC3 trinucleotide context of the edited sites in patients that could often resulted in sequence changes within some major histocompatibility complex (HLA in humans) restricted epitopes that can confer immune escape. Thus, we provide evidence that sub-lethal levels of APOBEC3 deaminases may expose the viral genome to beneficial mutations that influence HIV-1 adaptation and evolution in natural infection.

## Results

### Analysis of APOBEC3 evolving sites

To make predictions about the potential for APOBEC3 editing of the HIV-1 genome to influence virus diversification in natural infection, it was first necessary to carefully define the nucleotide sequence editing context preferences for the APOBEC3 proteins by titration in virus producing cells (**Table 1**). Vesicular stomatitis virus G (VSV-G) pseudotyped HIV-1 *vif*-deficient (HIV-1 pIIIB/Δvif) stocks produced in the presence of escalating doses of each of four human APOBEC3 genes that are the cellular targets for the HIV-1 protein Vif —namely, *APOBEC3D*, *APOBEC3F*, *APOBEC3G* and *APOBEC3H*—were therefore used to challenge cultured 293T cells.

**Table 1:** Single-cycle titration transfection conditions and mutation rates per conditions.

	Wild-type APOBEC3^a^^,^ b	Mutant APOBEC3^c^	Mutation Rate
**A3D**	A3D pcDNA3.1 (µg)	GFP vector (µg)	
	0	2	9.46×10^−4^
	0.02	1.98	1.17×10^−3^
	0.1	1.9	1.23×10^−3^
	0.5	1.5	1.62×10^−3^
	2	0	2.16×10^−3^
**A3F**	A3F pcDNA3.1 (µg)	mutA3F (E251Q)	
	0	3	1.03×10^−3^
	0.03	2.97	1.11×10^−3^
	0.1	2.9	2.09×10^−3^
	0.3	2.7	2.66×10^−3^
	1	2	3.34×10^−3^
	3	0	8.44×10^−3^
	0	3^d^	7.86×10^−4^
**A3G**	A3G pcDNA3.1 (µg)	mutA3G (E259Q)	
	0	1	7.45×10^−4^
	0.01	0.99	1.03×10^−3^
	0.03	0.97	1.73×10^−3^
	0.1	0.9	5.86×10^−3^
	0.33	0.67	2.01×10^−2^
	1	0	2.04×10^−2^
	0	1^d^	7.98×10^−4^
**A3H**	A3H pcDNA3.1 (µg)	pcDNA3.1	
	0	3	7.64×10^−4^
	0.03	2.97	1.66×10^−3^
	0.1	2.9	1.88×10^−3^
	0.3	2.7	3.41×10^−3^
	1	2	5.87×10^−3^
	3	0	9.29×10^−3^

a 3 µg of Vif deficient HIV-1 pIIIB/Δvif construct and 0.15 µg each of Vesicular Stomatitis Virus-G (VSV-G) envelope construct were co-transfected with wild type APOBEC3 expression vector and non-editing mutant APOBEC3 or empty vector in various conditions.

b Wild-type APOBEC3 construct in pcDNA3.1 expression vector.

c Non-editing mutant APOBEC3 or absent APOBEC3 construct.

d Empty pcDNA3.1 vector.

**Figure 1:**
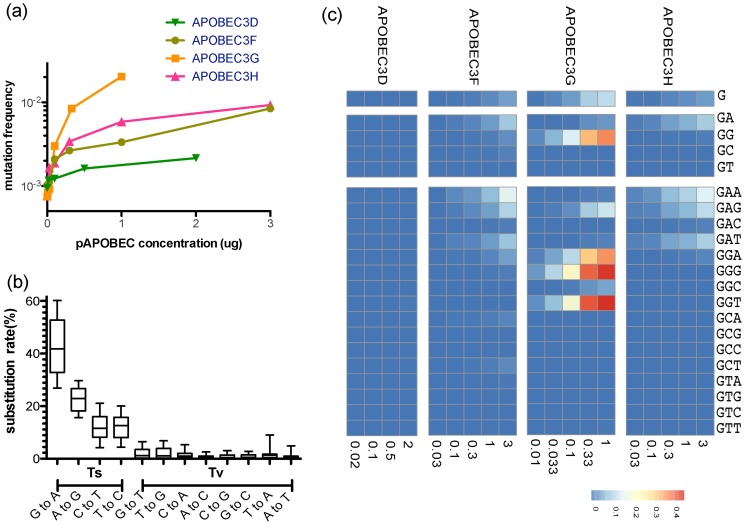
Site-specific editing frequencies in infected cells from single-cycle APOBEC3 titration transfection experiments. The single-cycle substitution rate for HIV-1 in the absence of human APOBEC3 was 8.6×10^−4^ mutations per nucleotide, whereas the mean single-cycle substitution rate for HIV-1 in the presence of human APOBEC3 ranged from about 1×10^−3^ to 2×10^−2^ per nucleotide substitution (**A**). The frequency of substitutions increased significantly in the region of the Gag gene of HIV-1 we sequenced in accord with increasing concentrations of the APOBEC3 proteins. The maximum single-cycle substitution rate for HIV-1 was 2×10^−3^ substitutions per site in the presence of APOBEC3D, 1.4×10^−2^ substitutions per site in the presence of APOBEC3F, 2.7×10^−2^ substitutions per site in the presence of APOBEC3G and 1.1×10^−4^ substitutions per site in the presence of APOBEC3H. The concentration of APOBEC3 at which we observed half of the estimated maximum substitution rate was 0.02 for APOBEC3D, 2.09 for APOBEC3F, 0.23 for APOBEC3G, and 0.22 µg for APOBEC3H. The single-cycle substitution rate for each mutation type (transition = Ts or transversion = Tv) of HIV-1 in the titration transfection experiments differed by 1.2 order of magnitude (**B**). For each of the human APOBEC3 proteins, we show the positions in the Gag gene of HIV-1 where the frequency of G-to-A mutation increased with increasing amounts of human APOBEC3 protein (Spearman rank correlation coefficient, *P*-value<0.05) (**C**). The G-to-A mutations are shown in a number of contiguous nucleotide sequence editing contexts. We used a sliding window to deduce the base frequency of G-to-A mutations (in each contiguous nucleotide context of the edited sites for each APOBEC3 protein (APOBEC3D, APOBEC3F, APOBEC3G, and APOBEC3H) using the total G-to-A mutation frequency at increasing concentration. Positions with a non-significant increase in G-to-A mutations were excluded from the calculations.

We screened for mutations in HIV-1 nascent retroviral cDNA with high throughput 454 pyrosequencing using a statistical framework to improve measurement accuracy. Barcoded oligonucleotide primers with unique molecular identifiers were used to amplify a PCR pool that was then sequenced with sufficient depth of coverage to redundantly cover the chosen portion of the viral genome. By stringent filtering and correcting the raw sequencing data, the platform efficiently reduces most sequencing errors generated during pyrosequencing [31]. We separated the resulting viral sequences by sample using the index sequence. Sequence alignments made to the HIV-1 pIIIB/Δvif reference sequence were optimized to reduce alignment errors introduced by insertions-deletions (indels) associated with the pyrosequencing chemistry, correct for incomplete extension miscalls, and filter out less abundant sequencing reads. We used a statistical analytical framework to filter the data for error correction and then built the haplotypes present in the viral populations. With this approach, we found a significantly lower average mutation frequency than the typical analysis of these data (average of 8.45×10^−4^) (**Figure 1A**), consistent with the estimations of others [2]. The nucleotide substitution rate for each mutation type (transition or transversion) differed by 1.2 orders of magnitude (**Figure 1B**). The different rates of nucleotide substitution likely reflect viral RT and RNA polymerase II fidelity and the asymmetrical substitution bias for faster accumulation of G-to-A mutations, the expected result of APOBEC3 editing. Random PCR amplification bias did not affect the reliability of the measurements [32].

**Figure 2:**
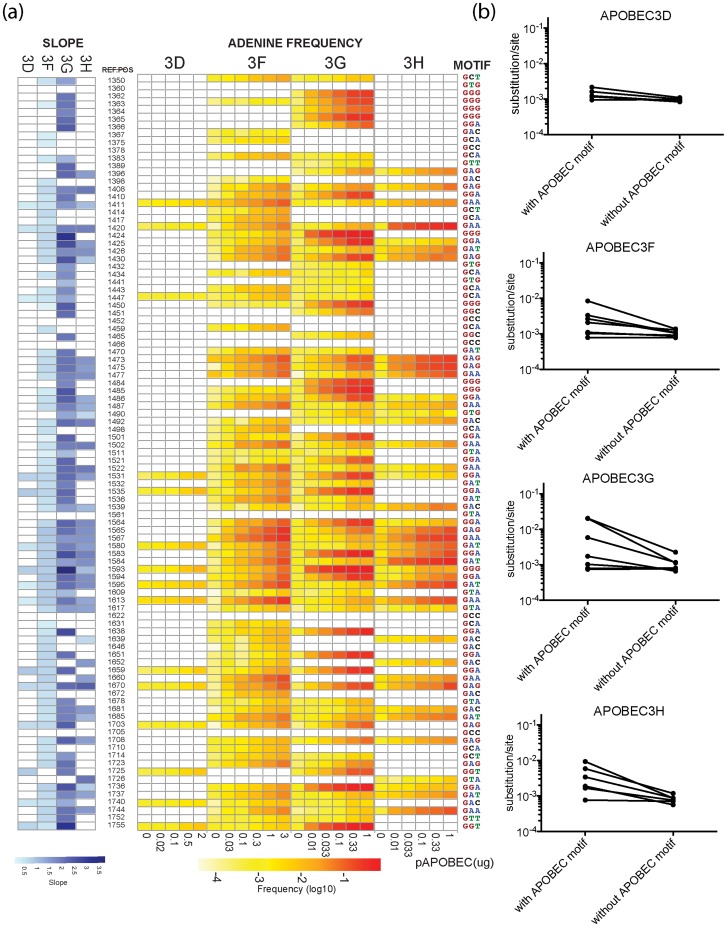
Positions of G-to-A mutations within the APOBEC3 trinucleotide context of the edited sites are shown in the HIV-1 genome. A heat map of the slope of the logistic regression of deamination frequency with increasing amount of APOBEC3 protein expression is shown at left (Spearman correlation coefficient, *P*<0.05) (**A**). The nucleic acid position in HXB2 (GenBank Accession number K03455) is to the right of the G-to-A position. A heat map of G-to-A frequency in APOBEC3 trinucleotide context of the edited sites with increasing amounts of expression of each APOBEC3 protein is shown in the middle panel. The location of the guanosine in the trinucleotide context of the edited sites in the segment of the targeted sequence is shown at right. Mutation frequency comparison with and without guanosines within the defined APOBEC3 trinucleotide context of the edited sites per each enzyme is shown (**B**). Upon removal of guanosine in the APOBEC3 trinucleotide context of the edited sites, substitution rate lowered to 9.77×10^−4^ mutations per nucleotide substitution.

Using these results, we identified G-to-A mutations in plus strand DNA as a genetic signature to identify *a posteriori* APOBEC3 nucleotide contexts of the edited sites. Each G-to-A mutation was considered independently (**Figure 1C**). Because there is about a two-fold increase in the frequency of adenosine relative to guanosine in the viral genome, we corrected for a bias in the 5′-GpA-3′ and 5′-GpG-3′ context raw numbers. For each of the four human APOBEC3 proteins that are the cellular targets for the HIV-1 protein Vif, we identified positions in the Gag gene region of HIV-1 we sequenced where the site-specific G-to-A substitution frequency increased significantly with increasing APOBEC3 protein abundance (Spearman *P*<0.05; **Figure 2A**).

The four APOBEC3 proteins had a clear bias for the plus strand 5′-GpG-3′ or 5′-GpA-3′ in the trinucleotide context of edited sites that can serve as signatures for specific *APOBEC3* gene activity (**Figure 1C**). APOBEC3G exhibited the highest frequency of G-to-A mutations in a 5′-GGD-3′ (where D is the IUPAC code for G, A, or T) and 5′-GAG-3′ context of edited sites and the most hypermutated sequences (**Figure 2A**). APOBEC3F and APOBEC3H proteins showed high frequencies of G-to-A mutation (range, 0.08 to 0.15) in both 5′-GAD-3′ and 5′-GGA-3′ contexts of edited sites. APOBEC3D showed a high frequency of a 5′-GGD-3′ context of edited sites, but the least activity of the four APOBEC3 deaminases tested under these experimental conditions.

For the trinucleotide context of edited sites within the Gag region of HIV-1 sequenced, G-to-A mutation did not invariably happen in all the potential APOBEC3 trinucleotide context of the edited sites. This observation suggests that other factors inherent to the sequence may affect the activity of these cytidine deaminases. The tryptophan (5′-UGG-3′) to a stop (5′-UAG-3′ or 5′-UAA-3′) codon change, for example, occurred at these two positions at a different frequency in the four APOBEC3 deaminases (**Figure S2**). Further, the 5′-GCC-3′, 5′-GCG-3′, and 5′-GTC-3′ trinucleotide contexts were not noticeably affected by any of the four APOBEC3 proteins. We did not find a statistically significant enrichment for the rare cytosine-to-thymidine (C-to-T) mutations brought about by a conflict between APOBEC3 editing and guanosine∶uridine (G∶U) mismatched base pair repair in regions of the genome where the plus-strand may become briefly single-stranded during reverse transcription [8].

In the absence of human APOBEC3 protein, the most commonly recovered mutations were random G-to-A or C-to-T transition mutations and −1 frameshift mutations from RT errors. The mutation rate during a single-cycle of HIV-1 replication was approximately 8.45×10^−4^ mutations per nucleotide (95% confidence interval [7.5, 9.6]×10^−4^ per nucleotide substitution). In the presence of increasing levels of human APOBEC3 protein, the mutation rate during a single-cycle of HIV-1 replication rose by a factor of between 2 (for APOBEC3D) and 20 (for APOBEC3G), which at sub-lethal levels would likely increase virus diversification and allow HIV-1 to evolve at different rates (**Figure 1A**) [33]. Once the guanosine in the trinucleotide context of edited sites was removed to correct for the substitution rate in the viral sequence, the average mutation rate corresponded to measurements for single-cycle of HIV-1 replication in the absence of functional APOBEC3 (**Figure 2B**). Thus, human APOBEC3 deaminase activity is evident in both a directional substitution bias and a higher substitution rate.

### Study subjects and viral sequencing

To explore the effects of APOBEC3 editing of HIV-1 genomes on virus diversification in patients, we produced longitudinal pyrosequencing data from the Gag and Vif genes of HIV-1 proviral DNA in peripheral blood mononuclear cells (PBMCs) isolated from ten patients during acute or early HIV-1 infection and at a second time point approximately 24 to 26 weeks later before the start of combination antiretroviral therapy. Table S1 shows the clinical characteristics of the ten patients. Transmission risk factors were not able to be determined in two patients (S002 and S006). The range of the estimated duration of infection was between 11 and 70 days [34]. Figure S2 shows the temporal changes in the mean levels of HIV-1 RNA in plasma (range, 779 to 57.6×10^6^ copies per ml) and CD4+ T-cell number counts (range, 311 to 760 cells per ml^3^). Table S2 shows the high-resolution HLA genotypes. A genetic screen of the *APOBEC3D*, *APOBEC3F*, and *APOBEC3G* genes found no single nucleotide polymorphisms associated with loss-of-function. The poorly expressed *APOBEC3H* hap I [35], [36] was found for seven patients, four of whom were homozygotes (S001, S005, S007, and S010) and three of whom were heterozygotes (S004, S006, S010; **Table S3**) [35]–[39].

To look for changes in the genetic structure of the HIV-1 population in each patient through time, we performed high depth gene sequencing with the aforementioned performance improvements for haplotype reconstruction to achieve the sensitivity and molecular resolution necessary for distinguishing among individual viral variants [31]. DNA isolated from patient PBMCs was subjected to HIV-1 Gag (nucleotide positions 977–1564 corresponding to HXB2) and Vif (nucleotide positions 5041–5619) gene sequencing using the 454 Life Sciences' GS-FLX pyrosequencing system. The median number of viral DNA template copies was 23,902 (interquartile range [IQR], 8,909 to 34,103) as measured by quantitative polymerase chain reaction (qPCR).

To estimate the virus population structure of the sample from the pyrosequencing reads, we aligned the reads to a consensus sequence and collapsed the repeated reads to build the haplotypes present in the viral population. We assembled the haplotypes in accordance with the informative de-noised reads of prescribed length such that the fewest haplotypes can account for the most reads. We then estimated the frequency of the reconstructed haplotypes present in the population. After error correction of the sequencing reads and reconstruction of the explaining haplotypes, the median number of the minimal inferred candidate haplotypes present in the population was 30.5 for the Gag (IQR, 5.75 to 67.0) and 35.65 for the Vif (IQR, 9.50 to 59.00) genes of HIV-1 (**Table S4**). The number of input molecules exceeded the fold depth of sequence data attained. This criterion is necessary to avoid bias caused by selective amplification and artifacts due to sampling and technical variability caused by pyrosequencing [40]. Binomial power calculations suggest that a sample size of 25,000 sequences gives a 96% likelihood of a variant present at 0.02% of the virus population to occur at least twice in the sample [24]. The depth of sequencing reads (range, 28,000 to 84,500) is sufficient to detect viral variants present at or above 0.02% of the population (**Table S4**).

### Phylogenetic analysis of viral sequences

**Figure 3:**
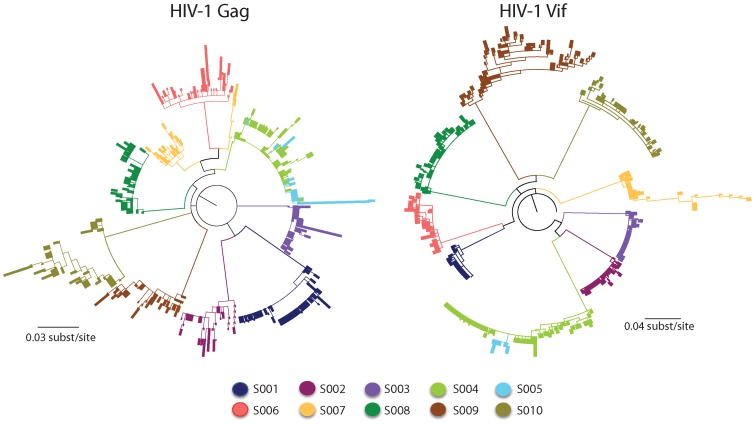
Maximum-likelihood tree for HIV-1 sequences from the patients studied. Shown are the maximum-likelihood phylogenies of viral nucleotide sequences from the Gag and Vif genes of HIV-1 derived from the ten patients at two time points during infection rooted with the estimated ancestral subtype B sequence [43]. The sequences from each patient are individually colored and labeled accordingly. The maximum-likelihood tree shows viral sequences that belong to a transmission pair (S004 and S005) and a dually infected patient (S007). Branch lengths indicate the number of nucleotide substitutions per site. The scale bar at the bottom refers to the degree of sequence mismatch. Maximum-likelihood bootstraps are presented below each branch.

Nucleotide sequences corresponding to the Gag and Vif genes of HIV-1 sampled at the early and late time points during infection were subjected to maximum-likelihood methods of phylogeny estimation. Figure 3 shows the maximum-likelihood trees of phylogenetic relationships among the aligned haplotype sequences of the Gag and Vif regions of HIV-1 from the ten patients. The topology of the tree shows distinct patient-specific clades, each with >95% of branch support [41], except for patient S007. Consistent with the short time since transmission and rapid expansion of virus from a distinct transmitted founder in the new host, the phylogenetic tree for patient S007 has short branch lengths and few internal branches.

A particular transmitted founder virus, which had been subjected to a stringent genetic bottleneck, successfully established HIV-1 infection in nine of the ten patients studied. For seven of the ten patients, the viral sequences formed distinct patient-specific monophyletic lineages, each with high statistical support (>99% probability). Viral sequences from a multiply infected patient (S007) did not coalesce at a single transmitted founder in the maximum-likelihood tree, consistent with more than one transmitted founder virus being responsible for establishing a productive infection. In two patients of known sexual congress (S004 and S005), each of who had a distinct transmitted founder virus, there was intermixing of viral sequences at the later sample time point, which were valid and did not result from cross-contamination of amplicons.

The *Highlighter* plots of viral sequences from each patient showed the random distribution of nucleotide polymorphisms across them consistent with a dispersal of variants that arise from a particular transmitted founder virus (**Figure S3**). Maximum diversity of sequences within the discrete viral lineages from the nine patients with a particular transmitted founder virus was low (mean 0.44%; range 0.15 to 0.73% and mean 0.53%; range 0.10 to 1.56% for the Gag and Vif genes of HIV-1, respectively). The maximum diversity of sequences from the patient with more than one transmitted founder virus (mean 1.38% and 1.87% for the Gag and Vif genes of HIV-1, respectively) exceeded that found in the viral sequences from the other patients.

### Analysis of evolving sites

**Figure 4:**
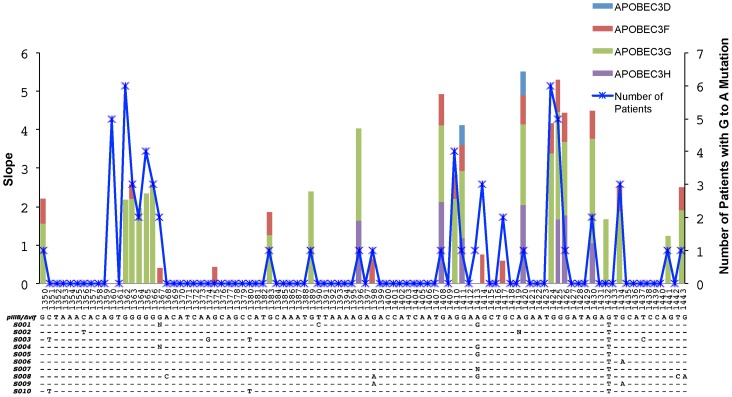
Overlap of the APOBEC3 edited HIV-1 genomes in cell culture experiments and in patients. A 96-base pair segment that overlaps a region sequenced in the Gag gene of HIV-1 in cell culture experiments and from patients is shown. APOBEC3 trinucleotide context analysis identified 26 sites that were significantly enriched for G-to-A mutations in more than one patient. The right y-axis corresponds to the number of patients and the left y-axis represents the slope of the logistic regression corresponding to the cell culture G-to-A mutation per concentration where significant correlation between these two values was observed (Spearman's test, *P*<0.05). The positions are numbered according to HXB2 and the alignment shows the HIV-1 pIIIB/Δvif sequence and the consensus sequence for each patient at the early time point. The blue line shows the number of patients with HIV-1 genomes that have significantly increased numbers of G-to-A mutations in positions that conform to the trinucleotide context of the edited sites and accrue over time (Fisher's exact test *P*<0.01). The light blue, red, green, and purple areas show the slopes with significant correlation (Spearman's test, *P*<0.05) for each G-to-A mutation with increasing concentrations of the APOBEC3D, APOBEC3F, APOBEC3G or APOBEC3H proteins, respectively.

Based on the evidence for trinucleotide contexts of edited sites for the APOBEC3 deaminases from titration transfection experiments, we examined the potential for APOBEC3 editing of HIV-1 DNA to contribute to adaptation and evolution in natural infection. Analysis of specific editing frequencies at individual guanosines for each of the four *APOBEC3* genes revealed a clear overlap with sequence changes observed in patients (**Figure 4**). Genomic context, such as adjacent nucleotides or local structural constraints, may have moderated against the effects of APOBEC3 editing at certain nucleic acid positions. Because the trinucleotide context of edited sites is shared among APOBEC3G, APOBEC3F and APOBEC3H, the two former cytidine deaminases could affect APOBEC3 editing for the seven patients who carried the less active form of APOBEC3H (haplotype I; homozygotes S001, S005, S007, and S010 and heterozygotes S004, S006, and S009; **Table S3**).

We next assessed whether APOBEC3 editing contributes to the genetic diversification of the virus populations in these patients. Viral sequences from the first time point differed from their respective consensus sequence by a median value of two (2) nucleotides in the Gag gene of HIV-1 and one (1) nucleotide in the Vif gene of HIV-1. At the second sampling time point 24 to 26 weeks later, the sequences from the Gag and Vif genes of HIV-1 differed from the particular consensus sequence by a median value of three (3) and two (2) nucleotides, respectively. We found relatively low frequencies of per nucleotide site insertions-deletions (indels) (1.47×10^−3^ and 9.42×10^−5^ for the Gag and Vif genes of HIV-1, respectively) and stop codons (2.44×10^−4^ and 1.03×10^−4^ for the Gag and Vif genes of HIV-1, respectively).

**Figure 5:**
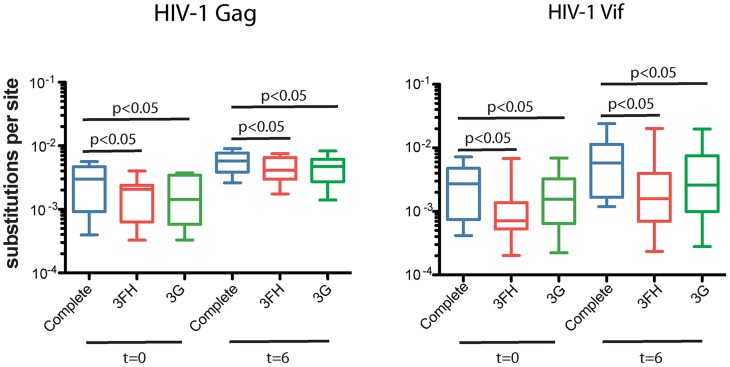
Pairwise distance analysis. Removal of the guanosine from the APOBEC3 trinucleotide context of the edited sites produced a significant decrease in the observed pairwise genetic distances of the sequences compared to the early time point consensus sequence, expressed as number of substitutions per site (*P*-values<0.05 are considered statistically significant).

The sequence diversity within each patient was calculated as the Hamming distance between sequences after weighting by the number of collapsed sequences and correcting for the sequence length. The frequencies of nucleotide substitutions measured across the sequenced regions gauges the proportion of nucleotide sites at which the viral sequences being compared are different (p-distance). We excluded the multiply infected patient (S007) and restricted our analysis to the nine patients with infection consistent with a single transmitted founder virus in which the observed sequence diversity is expected to be due to mutations that have happened after HIV-1 transmission. The maximum number of variable nucleotide sites within individual viral populations ranged from 0.6% (with mean diversity 0.3%) at the early time point to 0.9% (mean = 0.6%) at the later time point for the Gag gene of HIV-1 and 0.7% (mean = 0.3%) at the early time point to 2.4% (mean = 0.8%) at the later time point for the Vif gene of HIV-1 (**Figure 5**). After weighting by the number of collapsed sequences, the inter-sequence pairwise distances for the sequence sets at the early time point were significantly lower than at the later time point during infection (Wilcoxon sum rank test, *P*<0.05 for both Gag and Vif genes of HIV-1). The overall average ratio of nonsynonymous to synonymous nucleotide substitutions (dN/dS), estimated using the Nei-Gojobori algorithm as implemented in SNAP [42], were consistent with strong purifying selection over the time points sampled (HIV-1 Gag = 0.37, standard error of the mean [SEM] = ±0.07; and HIV-1 Vif = 0.23, SEM = ±0.04).

The overall mutation frequency among the nine patients, after correction for sampling time and frequency of the collapsed haplotype sequences with a maximum-likelihood analysis assuming a strict molecular clock, was estimated to be 3.8×10^−3^ and 6.5×10^−3^ per substitution per site per year for the Gag and Vif genes of HIV-1, respectively. When a Bayesian approach was taken and a strict molecular clock was used, the results obtained were very similar with estimated evolutionary rates of 4.1×10^−3^ and 4.2×10^−3^ per substitution per site per year for the Gag and Vif genes of HIV-1, respectively. Significantly, the viral sequences were sampled over a time period in which the evolutionary rate does not mirror a compound mutation and substitution rate. These values are consistent with the estimations of others [43]–[45].

To further assess the effects of APOBEC3 editing that happen at sub-lethal levels on the genetic structure of the HIV-1 populations, we analyzed the proportion of viral sequences with nucleotide changes happening in this way as well as their contribution to the genetic diversity of the viral populations. It follows that among the APOBEC3 trinucleotide contexts of the edited sites, which are distinguishable from the more random RT-induced G-to-A mutations, viral diversification should increase through acquisition of neutral or beneficial substitutions all the while circumventing the introduction of a deleterious stop codon or loss of an initiation codon. The frequencies of G-to-A mutations in the HIV-1 Gag gene sequences from the first to the second time point averaged: 31% (1.9/6.2 potential sites) for 5′-GGA-3′; 35% (3.1/8.5) for 5′-GGG-3′; 17% (0.5/2.8) 5′-GGT-3′; and 19% (1.2/6.5) for 5′-GAG-3′. In the HIV-1 Vif gene sequences, these frequencies averaged: 18% (1.6/8.9) for 5′-GGA-3′; 13% (1/7.8) for 5′-GGG-3′; 6% (0.3/4.8) for 5′-GGT-3′; and 15% (0.9/5.4) for 5′-GAG-3′.

To confirm that the APOBEC3 activity in virally infected cells may influence the substitution biases that could increase the substitution rate, we compared the pairwise genetic distance (nucleotide changes per site) with the complete alignments after removing the guanosine position from the APOBEC3 trinucleotide contexts of the edited sites identified in the cell culture experiments (5′-GAD-3′ and 5′-GGA-3′ for APOBEC3F and APOBEC3H and 5′-GGD-3′ and 5′-GAG-3′ for APOBEC3G) from the patients' collapsed alignments. Importantly, this process resulted in the pairwise distance between sequences being decreased significantly (Wilcoxon rank sum test *P*-value<0.05; **Figure 5**). Transition (purine to purine or pyrimidine to pyrimidine) and transversion (purine to pyrimidine or pyrimidine to purine) median ratio values were 4.66 (range 1.73 to 57.44) in the Gag and 5.34 (range 1.45 to 11.27) in the Vif genes of HIV-1. G-to-A (and C-to-T) transitions accumulated 5-fold faster than A-to-G (and T-to-C) transitions, an inequality in the evolutionary trajectory [32], [46]. In sum, these results demonstrate that stochastic or transient changes in APOBEC3 deaminase activity could have relevance for the directionality of HIV-1 evolution in natural infection.

### Analysis of immune escape facilitated by APOBEC3 editing

To determine whether natural selection acting on G-to-A mutations found in the APOBEC3 trinucleotide context of the edited sites could facilitate evasion of host immunity, we screened known or potential cytotoxic T lymphocyte (CTL) epitopes for positively selected sites. CTL epitopes had been established experimentally by interferon-γ enzyme-linked immunospot (ELISPOT) or predicted on the basis of amino acids that could serve as anchors to enable HLA binding or affect proteosome cleavage sites that abolish peptide binding, lessen T cell receptor recognition, or generate antagonistic CTL responses [47]–[49]. As an indicator of amount of natural selection operating on these CTL epitopes, we undertook a site-specific analysis of dN/dS in the Gag and Vif genes of HIV-1 using the Single Likelihood Ancestor Counting (SLAC) method implemented in HyPhy [50]. We focused on the G-to-A changes among the positions identified by reason of their significant selection signal (complete list and description in **Table S5**) and grouped them into positions appearing within or outside APOBEC3 trinucleotide contexts of the edited sites. We found an overrepresentation of positively selected positions within the APOBEC3 trinucleotide context of the edited sites in the Vif gene of HIV-1 (Fisher's exact test, *P* = 0.02; **Table 2**). Moreover, some of these positively selected positions in the Vif gene of HIV-1 appeared within a known or predicted HLA-appropriate epitope (S004 and S009, **Table S5**).

**Table 2:** Diversifying selection at individual nucleotide sites.

	HIV-1 Gag gene		HIV-1 Vif gene	
	Positive selection	Negative selection	Positive selection	Negative selection
APOBEC3 editing context	0	1	4	2
Non-APOBEC3 editing context	2	10	2	15
Total	2	11	6	17

A summary of the number of codon sites identified by the SLAC method implemented in by HyPhy [50] that show positive or negative selection at the APOBEC3 and non-APOBEC3 motifs in the regions of the Gag and Vif genes of HIV-1 we sequenced (*P*-value<0.02). The Vif gene of HIV-1 was over-represented with G-to-A mutations in an APOBEC3 editing context at positively selected sites (**Table S5**).

HIV-1 sequences encoding variants that could result in a lower predicted peptide binding score which would potentially confer a diminished or immune escape phenotype, were found within a HLA-appropriate epitope in the Gag (19 of 42; per patient range, 0 to 6) or Vif genes of HIV-1 (37 of 99; per patient range, 0 to 4) at the later time point during infection (**Tables 3** and **S6**). Most epitope escape mutations were found in those patients that carry HLA-A01:01:01, HLA-A02:05:01 or HLA-A03:01:01 or HLA-B07:02:01, HLA-B08:01:01 or HLA-B57:01:01 (S001, S002, S003, S009, and S010). Of the sites under positive selection, 7 of 19 sites in the Gag and 8 of 42 sites in the Vif genes of HIV-1 were a result of G-to-A mutations in APOBEC3 editing contexts. Clusters of G-to-A mutations in known or predicted HLA-appropriate epitopes were higher in some patients (S001 and S009) than in others (S002, S003, and S010). We found statistically significant evidence for G-to-A mutations in APOBEC3 trinucleotide contexts of edited sites that cause nonsynonymous substitutions in the amino acid residues located at the epitopes (Fishers exact test *P*<0.05). These data demonstrate that APOBEC3-induced mutation embedded in the HLA-restricted epitopes can accumulate over time as a consequence of immune selection pressure.

**Table 3:** CTL epitope variants found in the Gag and Vif genes of HIV-1 from the ten patients.

	HIV-1 Gag gene	HIV-1 Vif gene
Total escapes (epitope variants)	19	42
APOBEC3 mediated escape	7	8
No APOBEC3 mediated escape	12	34
APOBEC3 editing context	7	8
Overcome	0	5
Remain minority	3	1
Appear minority	0	2
Lost	4	0
no APOBEC3 editing context	12	34
Overcome	1	10
Remain minority	3	1
Appear minority	5	18
Lost	3	5
Stop codon introduced	6	0
APOBEC3 mediated escape	6	0
No APOBEC3 mediated escape	0	0

To identify G-to-A mutations in APOBEC3 trinucleotide contexts of edited sites in known or predicted HLA-appropriate epitopes in relation to the most common haplotype in the first time point in each patient, we compared the epitopes at both early and late time points during infection. At the second time point during infection, G-to-A mutations were preferentially found at 1/13 sites in the Gag and 6/23 sites in the Vif gene of HIV-1 (**Table S6**). Though only direct experimental studies can establish which of the G-to-A mutations are associated with the evasion of host immunity, we infer that many of these are positively selected sites at low frequency variants at the first time point that transition to fixation at the second. In this manner, APOBEC3 editing can affect the crucial interaction between the virus and the host during the earliest stages of infection, and thereby potentially influence the natural history of HIV-1 infection.

## Discussion

In this study we define at unprecedented depth the specific APOBEC3 trinucleotide contexts of edited sites in cell culture experiments and show that the equivalent mutations that accrue in viral DNA in cells from patients through time provide a source of genetic variation upon which natural selection acts; thus, resolving the widely debated contribution of APOBEC3 editing to the genetic changes underlying the evolution of HIV-1 populations in natural infection [13], [22], [33]. Using a statistical framework that detects and corrects pyrosequencing errors, we show that *APOBEC3D*, *APOBEC3F*, *APOBEC3G*, and *APOBEC3H*, the cytidine deaminases of the human APOBEC3 gene family that are the cellular targets for HIV-1 protein Vif, have distinct, but overlapping trinucleotide contexts of the edited sites associated with antiviral defense. Mapping these APOBEC3-mediated G-to-A mutations onto the viral sequences from ten patients with primary HIV-1 infection through time is informative of the impact that these human genes can have on virus diversification. The over-representation of G-to-A mutations in the viral sequences compared with A-to-G or C-to-T or T-to-C mutations (in a A-rich, C-poor genome) suggests that accumulation of APOBEC3 mutations is well tolerated in diversifying sites and could account for the skewing of nucleotide and codon usage in the viral genome. We note that the total number and location of the APOBEC3 trinucleotide context of the edited sites within the viral genome and the extent to which they can accumulate through time need to be accounted for in evolutionary inference at the population-level.

The tandem array of the seven human cytidine deaminases of the APOBEC3 gene family on chromosome 22, which we distinguish by their target sequence consensus, suggest that multiple, related antiviral functions can contribute to the control of virus infection. Differences in single-stranded DNA binding, as well as translocation along engaged templates, may explain the sequence specificity of APOBEC3 activity and processing accuracy [51], [52]. Nucleotides adjacent to the APOBEC3 editing context likely influence the kinetics of G-to-A mutation. Functional biases in cytidine deaminase activity suggest that people may differ in the predominant expression of APOBEC3 and that these functionality-related genes may play a role in the spectrum of innate resistance that protects against invading viruses and contributes to phenotype. This conclusion, which could apply to other types of viruses or retroviral elements, suggests that human APOBEC3 proteins have clear impact at the boundary between the virus and its host.

It has been posited that limiting-levels of APOBEC3 activity could result in lethal mutations rather than rapid adaptation through acquisition of neutral or potentially beneficial mutations [33]. Further, that a single incorporated APOBEC3 unit is likely to cause extensive and inactivating levels of HIV-1 hypermutation. These conjectures are based on *in silico* analyses of optimized reference sequences that would be estimated to account for the mutation levels of 39 near-full length patient-derived hypermutated viral sequences selected from the HIV Sequence Database. As the analyses began with highly mutagenized HIV-1 genomes with 5′-GpG-3′ or 5′-GpA-3′ signatures of APOBEC3 editing from which the non-hypermutated reference sequences were derived, the estimated effect is distorted by a clear selection bias. Studies that have original patient-derived non-hypermutated reference sequences clearly corroborate the relevance of small increases in mutation frequency affected by APOBEC3 for genetic changes underlying virus evolution [53].

Natural selection during virus infection can create advantageous mutations or eliminate deleterious ones. The rate of fixation of advantageous mutations, which is faster than the rate of fixation of neutral mutations, increases with the strength of selection. We found statistically significant evidence for positive selection acting on the Vif region of HIV-1 in the APOBEC3 trinucleotide context of the edited sites. Even though blockade by the HIV-1 protein Vif effectively counters the action of certain human APOBEC3 proteins that could lead to the lethal accumulation of mutations, some G-to-A mutation is produced that can increase genetic diversity and facilitate adaptation; an apparent shortcoming of APOBEC3 editing that should caution against the use of a HIV-1 Vif antagonist as a virus inhibitor.

When the trinucleotide context of the edited sites rests within an HLA-appropriate epitope so that G-to-A mutation affects peptide binding or T-cell antigen receptor recognition, the APOBEC3 proteins could be an important driver of mutations that enable a virus (of reduced replicative fitness) to evade host immunity [27]. G-to-A mutations within APOBEC3 trinucleotide contexts of the edited sites may be constrained, however, by pressure to retain possible useful structural elements or functional sites [54]. We show here that a number of diversifying codon sites in an APOBEC3 trinucleotide context, as indicated by the accumulation of non-synonymous nucleotide changes, were clustered within a number of HLA-restricted epitopes that could act as anchors for HLA binding or in the proximal three amino acid regions that could affect peptide processing. These data reveal that APOBEC3 can generate viral mutations in immune-susceptible locations that are subjected to strong positive selective pressure during the acute phase of infection.

Our analyses provide strong statistical evidence for an association between G-to-A mutation rates and HIV-1 diversification in natural infection. Consistent with APOBEC3 evolutionary footprints in the viral genome, we find a higher frequency of mutations in APOBEC3 than non-APOBEC3 edited sites introduced during sequential generations of HIV-1 within patients. The sub-lethal APOBEC3 editing that make subtle contributions to viral sequence diversity can lead to mutational fitness effects that should facilitate host adaptation, having been associated with the evasion of host immunity and evolution of resistance to antiretroviral drugs [21], [55]. The longitudinal analysis of HIV-1 infection in these ten patients gives important new insight into the causes and consequences of virus diversity upon which selection can act. The findings described here, therefore, suggest that the genetic conflict caused by the APOBEC3 innate immune effectors is an important determinant in explaining the mutational dynamic and directionality that underlies HIV-1 evolution.

## Methods

### Cell culture and creation of VSV-G-pseudotyped HIV-1 stocks containing APOBEC3D, APOBEC3F, APOBEC3G, or APOBEC3H

293T cells, which express little or no endogenous APOBEC3, were cultured in Dulbecco modified Eagle medium (DMEM) supplemented with 10% fetal bovine serum plus penicillin-streptomycin. Sub-confluent monolayers of 293T cells seeded in 35-mm plates were co-transfected with 3.0 µg of the *vif*-deficient HIV-1 pIIIB/Δvif construct, a vesicular stomatitis virus G (VSV-G) protein expression vector, and between 0.01 µg and 3 µg of pcDNA3.1-based expression vectors for APOBEC3D, APOBEC3F, APOBEC3G or APOBEC3H (haplotype II) using polyethylenimine (PEI; **Table 1**). Forty-eight hours later, viral supernatants were harvested, treated with 20 U/ml RQ1 DNase (Promega) in 10 mM MgCl_2_ for 3 h at 37°C, and then purified by pelleting through a sucrose cushion. Virus was quantified by a HIV-1 p24 Gag enzyme-linked immunosorbent assay (ELISA; Perkin-Elmer).

### Single-cycle HIV-1 infection and analysis of 293T cell DNA

Sub-confluent layers of 293T cells were infected with VSV-G-pseudotyped virus stocks equivalent to 50 ng HIV-1 Gag p24. The input virus was removed four hours later, and the cells were thoroughly washed before the addition of fresh medium. Two days after infection, the supernatant was harvested for quantification by HIV-1 Gag p24 ELISA. Viral infections were determined in single-cycle assays as described [56]. Total genomic DNA was isolated with the QIAamp DNA cell mini kit (Qiagen) and purified DNA was digested with *Dpn* I to remove any residual plasmid DNA. To normalize the input amount of viral DNA for sequencing using the 454 Life Sciences' GS-FLX pyrosequencing system (Roche), we measured the amount of the HIV-1 Gag gene DNA by qPCR as described [21]. The relative amount of HIV-1 target DNA was normalized to the quantification cycle for a concentration calibrator by using an external standard curve of serial 10-fold dilutions of a reference Gag gene of HIV-1 DNA.

### Study subjects

Over a period of up to 26 weeks, we tracked changes in the nucleotide sequences from the Gag and Vif genes of HIV-1 in peripheral blood sampled from ten infected patients. All ten patients had confirmed HIV-1 infection, were enrolled in a study of early HIV-1 infection. Acute HIV-1 infection was defined by the presence of HIV-1 RNA in plasma and a negative or weakly positive HIV-1 ELISA followed by a positive one. Early HIV-1 infection was defined by the presence of a positive HIV-1 ELISA confirmed by a detuned negative HIV-1 ELISA. All participants in the study had symptoms compatible with the acute retroviral syndrome and were treated with a combination of potent antiretroviral drugs (one protease inhibitor and two nucleoside reverse transcriptase inhibitors) within a median of 2 years (range, 1.5 to 3 years) from the time of diagnosis. Clinical and laboratory data and sample collection begins at enrollment and at prescribed interval study visits thereafter.

### Ethics statement

All patients provided written informed consent according to the guidelines of the Human Subjects Protection Committee of the University of California, San Diego. The University of California, San Diego Institutional Review Board approved the study.

### Extraction and quantification of target DNA

Genomic DNA was isolated from frozen PBMC samples (approximately 2 million cells) using the QIAamp DNA blood mini kit (Qiagen) according to manufacturer protocol. DNA was eluted in nuclease-free water (100 ul) and stored at −80°C until use. The amount of HIV-1 DNA was measured by qPCR of the Gag gene of HIV-1 with the TaqMan Universal PCR Master Mix (Applied Biosystems) on the 7900HT sequence detector (Applied Biosystems) as described [21]. The relative amount of HIV-1 target DNA was normalized to the quantification cycle for a concentration calibrator by using an external standard curve of serial 10-fold dilutions of reference HIV-1 linear full-length plasmid DNA derived from the pNL-43 plasmid. The amount of input cell DNA was normalized to the amount of human *CCR5* amplified using the forward primer CCR5-F 5′-ATCGGAGCCCTGCCAAAA-3′, the reverse primer CCR5-R 5′-TGAGTAGAGCGGAGGCAGGAG-3′, and probe CCR5-P 5′-FAM-CGGGCTGCGATTTGCTTCACATTG-BHQ-3′. All reactions were performed in quadruplicate.

### High resolution HLA typing

High resolution HLA genotyping was performed by next generation sequencing of exonic amplicons using the 454 Life Sciences' GS-FLX pyrosequencing system (Roche) with Conexio Assign ATF 454 software as described [57].

### APOBEC3H genotype and haplotype analysis

We performed genotype and haplotype analysis of APOBEC3H for previously identified variants that cause the amino acid polymorphisms R18L, G105R, K121D, and E178D by means TaqMan SNP Genotyping assays with an Applied Biosystems 7500 Real-time PCR detection system. For the N15Δ, K121D, K121N, and K121E polymorphisms, primers were designed to produce a PCR-product DNA that could be sized and sequenced. First round PCR was performed with A3H_EK2852F (5′-AGGCAGGAGAATCGCTTGAACTTG-3′) and A3H_EK4571R (5′-CCTCCCGGGTGGTGTCAGAT-3′) to amplify exon 1 and exon 2 for 30 cycles (94°C-30 sec; 58°C, 30 sec; 72°C, 1.5 min). For 121 polymorphisms, PCR-product DNA was diluted and directly sequenced with A3H_EK4112F (5′-CCCCTGCTCCTCCTGTGCCT-3′) and A3H_EK4522R (5′-CTTCCTGGCCTCCCACAGACC-3′). For N15Δ polymorphism, primers A3H_EK3068F-FAM (5′-FAM-ACAGCCGAAACATTCCGCTTACAG-3′) and A3H_EK3204R (5′-TTGTTTTCAAAGTAGCCTCTCGTGGG-3′) were used with Taq polymerase for initial denaturing for 2 min at 94°C, followed by 30 cycles (94°C, 15 sec; 58°C, 1 min) and a final 5 min extension at 72°C. DNA fragment analysis was performed on an Applied Biosystems 3730xl DNA Analyzer with 36 cm capillary array and analyzed by GeneMapper 4.0 software (Applied Biosystems/Life Technology).

### Viral DNA amplification, library preparation and pyrosequencing

To facilitate quantitative sampling of the viral population, we performed viral DNA amplification by PCR using high template volume, low cycle numbers, and multiple replicates that were pooled for sequencing. PCR primer design was predicated upon the alignment of multiple sequences from the HIV Sequence Database [58] to minimize biased amplification of the target DNA. We selected highly conserved regions in the Gag and Vif genes of HIV-1 that encompassed the APOBEC3 trinucleotide context of edited sites and known or predicted epitopes, which were an appropriate distance apart and within the read-length limits of the 454 sequencing technology employed. Primers design considered preexisting alignment covering the region of interest. Conserved primer locations were selected based on alignment positional entropy. Within the selected conservative sequences, we used degenerative bases for the APOBEC3 editing context. Unique molecular identifiers that label individual molecules in the pool moderate against erroneously attributing multiple identical sequences to low viral diversity and allelic skewing by biased PCR amplification.

Viral DNA isolated from 293T cells was amplified using the HIV-1 NL4-3 Gag gene-specific degenerate forward primer gag_F1329dg (5′-CGTATCGCCTCCCTCGCGCCATCAG [fusion primer A]- [multiplex identifier sequence (MID)]-CCCCACAARATTTAAACACCAT-3′, corresponding to positions 1328→1349) and reverse degenerate primer gag_R1785dg (5′-CTATGCGCCTTGCCAGCCCGCTCAG [fusion primer B]- [MID]-GTYTTACAATYTGGGTTYGCAT-3′, corresponding to a 1784→1763 reverse complement) to generate 457 bp of the Gag gene of HIV-1 (HXB2 genome 1328→1784).

Viral DNA in PBMC samples from patients was amplified using the HIV-1 Gag gene-specific forward primer A-Gag_977F_degEK (5′-primer A-GCTACAACCAKCCCTYCAGACAG-3′, corresponding to positions 977→1000) and the reverse primer B-Gag_1564R_degEK (5′-primer B-CTACTGGGATAGGTGGATTAYKTG-3′, corresponding to a 1564→1541 reverse complement) to generate 588 bp of the Gag gene HIV-1 (HXB2 genome 977→1564) or the HIV-1 Vif gene-specific forward primer A-Vif_5041F-EK innerF (5′-primer A-ATGGAAAACAGATGGCAGGTG-3′, corresponding to positions 5041→5061) and the reverse primer B-Vif_5623R-EK_innerR (5′-primer B-AGCTCTAGTGTCCATTCATTGTATG-3′, corresponding to a 5623→5599 reverse complement) to generate 583 bp of the Vif gene of HIV-1 (HXB2 genome 5041→5623).

PCR was performed using the High Fidelity Platinum Taq DNA Polymerase (Invitrogen) with thermal cycling conditions of 94°C for 2 mins, followed by 35 cycles of 94°C for 15 sec, 54°C for 15 sec, 68°C for 1 min, with a final extension step at 68°C for 5 mins. DNA amplicon libraries were resolved on a pre-cast 2% agarose gel and purified with QIAquick Gel Extraction kit (Qiagen) and AMPure XP SPRI beads (Beckman Coulter Inc.). To determine amplicon library quality, a Bioanalyzer (Agilent) was used and quantity for amplicon samples along with KAPA Library Quant Kit (KAPA Biosystems). An equimolar mix of the amplicon libraries was subjected to emulsion PCR and DNA sequencing using the 454 Life Sciences' GS-FLX pyrosequencing system (Roche). Multiplex identifiers were used to bin the sequence reads before analysis.

### Sequence clean up and assembly

As the quality of sequence may decrease across a sequence read, we quality filtered the sequence data before analysis [31]. Each sequence read had to pass a series of standard metrics to ensure the output of high quality sequence reads while maintaining the maximum possible length of the output sequences. We excluded sequences that had a frameshift relative to a reference sequence, were too short, contained long direct repeats, were a recombinant, or had a close pair of matched-length indels that created a short compensated frameshift. In this analysis, we used the same methods and parameters for obtaining a clean alignment as in previous work [31]. Viral sequences that were observed only once were also excluded to further reduce technical noise. After k-mer mapping, reads were pairwise aligned to the consensus template pIIIB/Δvif reference sequence for the cell culture experiments, taking into account data-specific indels, and thereby reducing dependency on a generic template.

We aligned the viral sequences obtained from peripheral blood from patients by Segminator II (version 0.1.1) using the HXB2 sequence (GenBank accession number K03455) as a reference for assembly [31], [59], [60]. We generated a consensus sequence for each patient, which was then used as a reference sequence for the patient-specific re-alignment. We used a statistical model that accounts for site-specific error rates to separate errors from true variations and remove chimeric molecules that arise from PCR or pyrosequencing errors by applying the Predator algorithm default implemented in Segminator II. This statistical framework maintains the reading frame and corrects for length errors in homopolymeric runs of nucleotides, the characteristic error of 454 pyrosequencing. Nucleotide sequence alignments used in this study were deposited in GenBank with the accession numbers (KJ016272–KJ017738).

### Nucleotide context of the edited sites

We performed a tally of G-to-A mutations among the viral sequences from the titration transfection experiments that contain a guanosine within a binucleotide, trinucleotide, tetranucleotide, or hexanucleotide context of the edited sites for the HXB2 reference sequence using a sliding window. Each G-to-A mutation was considered independently. A tally of C-to-T mutations was used to assess the extent of noise in the analysis. The viral sequences were treated as character arrays, and therefore each trinucleotide context of the edited sites was compared separately with the same location in the reference sequence.

For each position, we separately checked for the fraction of mutations increasing with the concentration of APOBEC3 by calculating the significance of the Spearman rank correlations, without correcting for multiple testing. Only those positions that changed significantly with increasing concentrations of the different enzymes for both forward and reverse reads were chosen as possible APOBEC3-induced G-to-A change. Motifs that were statistically significantly overrepresented in this dataset were designated as APOBEC3 trinucleotide editing contexts and used in further analyses. We did not find any significant associations with longer motifs. The strength of the effect was evaluated by calculating the slope from a logistic regression of the mutation probability against the concentration of the enzyme.

To analyze APOBEC3 editing in patients, we calculated the sequence divergence for each position in the Gag and Vif genes of HIV-1 and determined base frequencies at each position in the alignment. We analyzed the G-to-A mutations in the defined APOBEC3 trinucleotide editing contexts that increased significantly between the two time points. We used the previously cleaned alignments to calculate the frequency of G-to-A mutations at each position with guanosine in the early consensus sequence by using an in-house Perl script. These ratios were compared between time points by Fisher's exact test and G-to-A mutations increasing in the later time point with a *P*-value<0.01 were taken into account for further analysis.

### Analysis of HIV-1 evolution in patients

To avoid using uninformative sequence repeats, the viral sequences were collapsed into shared non-recombinant haplotypes representing only unique sequences using the tools in the FASTX-toolkit (version 0.0.13) implemented in Galaxy (https://main.g2.bx.psu.edu). The collapsed haplotypes in each patient were realigned using the alignment method implemented in MUSCLE (version 3.8.31) [61]. We kept sequences corresponding to variants present above 0.2% of the total existing variants in the collapsed alignments. The best-fit model of nucleotide substitution was estimated for each of the collapsed alignments with a maximum-likelihood method using PhyML version 3.0 as implemented in jModelTest2 [62]. We constructed maximum-likelihood trees for the Gag and Vif genes of HIV-1 with and without the APOBEC3 trinucleotide context of the edited sites for each patient. Because maximum-likelihood tree error may increase when unreliable sites are included, we estimated trees on viral sequence sets from which gaps in the alignment were removed and considered as missing data. Ancestral states of each node of the trees constructed with the complete alignments (including the trinucleotide context of the edited sites) were estimated by a maximum-likelihood method using PhyML version 3.0 [62] applying approximate likelihood ratio test (aLRT) for branch support [41]. Nucleotide sequences from all ten infected patients were analyzed with phylogenetic trees using the neighbor-joining method together with the *Highlighter* sequence visualization tool (www.HIV.lanl.gov) to trace commonality between sequences in an alignment based on individual nucleotide changes.

To assess the type of evolutionary forces operating on the patient derived viral sequences, we estimated the overall ratio of dN/dS using Synonymous Non-synonymous Analysis Program (SNAP) (www.HIV.lanl.gov) [42]. Site-specific analyses of dN/dS were undertaken using the SLAC method implemented in HyPhy at the Datamonkey webserver (http://www.datamonkey.org/) [50], [63]. We conducted this analysis with the general reversible (REV) model of nucleotide substitution on phylogenetic trees using the neighbor-joining method (cut-off *P*-value = 0.1). We selected the G-to-A changes among all the identified positions under selection and then analyzed their possible association with APOBEC3 activity by testing whether there was an overrepresentation of G-to-A mutations in an APOBEC3 editing context.

The mutation rates were estimated from the maximum-likelihood phylogeny of viral sequences collected at the early and late time points during infection. To account for the time-dependency of evolution rate estimates, we rooted the tree at a position most compatible with a strict molecular clock and analyzed the slope of the regression of root-to-tip distances against the dates of sampling with Path-O-Gen version 1.4 (http://tree.bio.ed.ac.uk/software/pathogen/). Evolutionary rates obtained using this maximum-likelihood method were confirmed by a Bayesian approach using phylogenetic reconstructions of the complete alignments of the ten most represented haplotypes for each patient sampled at early and late time points during infection. This analysis was performed using BEAST version 1.7.4 [64] with a GTR + gamma evolutionary model assuming a strict molecular clock and a constant population size. We computed the posterior probability of the model to obtain the Bayes factors to discriminate among the models.

### Genetic distances

We calculated the number of substitutions per site, performing a pairwise comparison of every sequence to the first time point consensus for viral sequences from each patient's collapsed alignments using MEGA (version 5.2.2), weighting the obtained values with the number of sequences of each haplotype, and then determining the weighted average of each time point. Two alignments of the collapsed haplotypes in the sequenced populations were generated: One without the sites containing guanosine in the 5′-GAD-3′ and 5′-GGA-3′ trinucleotide contexts in the first time point consensus (corresponding to APOBEC3F and APOBEC3H editing), and the other without the sites containing guanosine in 5′-GGD-3′ and 5′-GAG-3′ trinucleotide context (corresponding to APOBEC3G). We removed the guanosines on APOBEC3 trinucleotide contexts of the edited sites from the alignments using the Unix stream editor command ‘sed’. Pairwise distances of the complete alignments in each time point were compared with the ones obtained in the absence of guanosine in the trinucleotide context of the edited sites using a Wilcoxon signed-rank sum test (*P*<0.05). Values were normalized with their respective sequence length prior to comparisons.

### Analysis of escape mutations within HLA class I-restricted epitopes

We genotyped the six-digit HLA types using a next-generation sequencing method as described [65] (**Table S2**). To investigate whether the CTL epitope variants or escape mutations were mediated by APOBEC3 activity, we retrieved known or predicted MHC class I-restricted epitopes from the HIV Molecular Immunology Database [66] (http://www.hiv.lanl.gov/content/immunology/variants/variant_search.html).

### Statistical analysis

Standard descriptive statistics were performed with the use of the STATA, GraphPad or R packages (version 1.1-1, http://CRAN.R-project.org/package=binom) [67], [68].

## Supporting Information

Figure S1 **Frequencies of tryptophan to stop codon changes in viral sequences in infected cells from titration experiments.** Tryptophan (5′-UGG-3′) to stop codon (5′-UAG-3′ or 5′-UAA-3′) happened at different frequencies at two different positions. G-to-A mutation did not invariably happen in the APOBEC3 trinucleotide context of the edited sites, suggesting that other factors may affect cytidine deaminase activity.
(TIFF)

Figure S2 **Patient characteristics.** Shown are the mean levels of HIV-1 RNA in plasma and CD4+ T-cell number counts for the samples from the ten patients.
(TIFF)

Figure S3 **Neighbor joining phylogenetic trees and** ***Highlighter*** **plots of viral sequences from patients.** Shown are *Highlighter* plots and neighbor-joining trees for the Gag and Vif genes of HIV-1 for all patients. Shown are the first (Day 0, green letters) and second (25 weeks, purple letters) time points during infection. The numbers present the number of collapsed sequences. The APOBEC3-mediated mutations are highlighted with lavender dots. Most of the evolved variants sampled at the later time points carry G-to-A mutations in the APOBEC3 trinucleotide context of the edited sites. The nucleotides that do not match with the master are assigned a color as given (A: Green; T: Red; G: Orange; C: Light blue; IUPAC codes (as regular characters): Dark blue; Gaps: Gray; Circle: APOBEC3, Diamond: G-to-A).
(PDF)

Table S1 **Patients' characteristics and estimated date of infection based on the onset of acute retroviral symptoms.**
(DOCX)

Table S2 **High-resolution HLA genotypes for the patients studied.**
(DOCX)

Table S3 **APOBEC3H haplotypes for the patients studied.**
(DOCX)

Table S4 **Number of sequence reads in the process of error correction and reconstruction of haplotypes.** After error correction, the reads were aligned and trimmed to encompass the greatest coverage, then collapsed into unique haplotypes. The median number of the minimal inferred candidate haplotypes present in the virus population.
(DOCX)

Table S5 **Positively and negatively selected codon sites in the Gag and Vif genes of HIV-1.**
(DOCX)

Table S6 **CTL epitopes found within the Gag and Vif genes of HIV-1.** Epitopes that are known or predicted on the basis of amino acid positions that could act as anchors for HLA binding (position 2 or 9) or could affect peptide processing were found through a search for the epitope variant and escape mutations deposited in the HIV Sequence Database (http://www.hiv.lanl.gov/content/immunology/variants/variant_search.html). Each patient's restricting HLA alleles and the optimal defined or predicted epitopes sequences plus three flanking amino acids are listed from both early (green) and late (orange) time points. The most common epitope sequences in early time points are listed on the top with light green shade. The dashed lines represent the same amino acids with the most common epitope sequence in the early time point and the changed amino acids are listed. The frequency changes greater than 20% were highlighted with red fonts. ^a^The position refers to corresponding position in the HXB2 sequence. ^b^Each patent's restricting HLA alleles are listed. ^c^The numbers reflect the frequencies of the epitope sequences in deep sequences in total reads.
(XLSX)
