# Supplementary figures and images for: Human APOBEC3 Induced Mutation of Human Immunodeficiency Virus Type-1 Contributes to Adaptation and Evolution in Natural Infection
Source: PLoS Pathog. 2014 Jul 31;10(7):e1004281. doi: 10.1371/journal.ppat.1004281 (PMC4117599; doi:10.1371/journal.ppat.1004281)

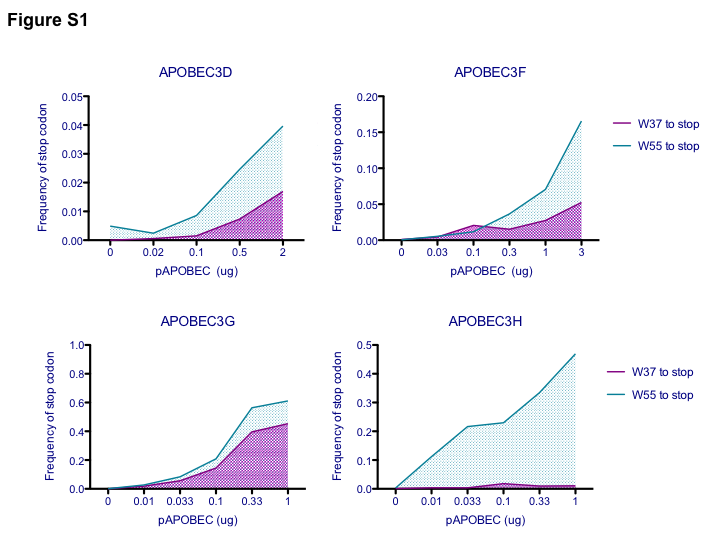

Supplement: Figure S1 — Frequencies of tryptophan to stop codon changes in viral sequences in infected cells from titration experiments. Tryptophan (5′-UGG-3′) to stop codon (5′-UAG-3′ or 5′-UAA-3′) happened at different frequencies at two different positions. G-to-A mutation did not invariably happen in the APOBEC3 trinucleotide context of the edited sites, suggesting that other factors may affect cytidine deaminase activity. (TIFF) [file ppat.1004281.s001.tiff]

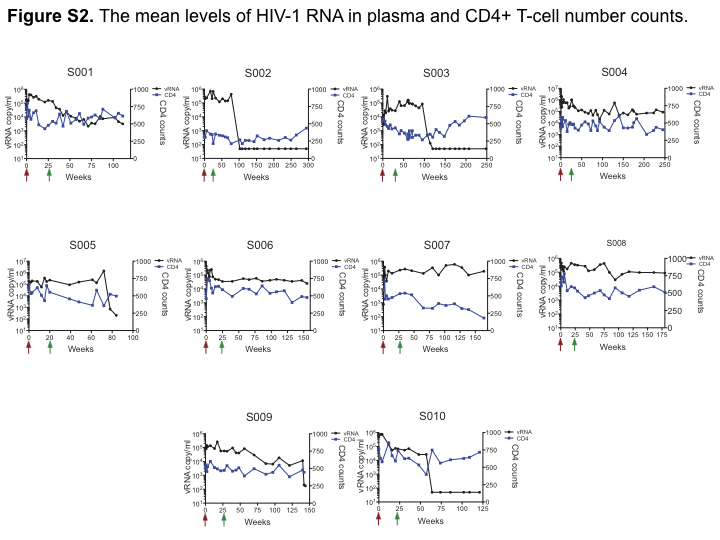

Supplement: Figure S2 — Patient characteristics. Shown are the mean levels of HIV-1 RNA in plasma and CD4+ T-cell number counts for the samples from the ten patients. (TIFF) [file ppat.1004281.s002.tiff]
