# Supplementary material for: Human APOBEC3 Induced Mutation of Human Immunodeficiency Virus Type-1 Contributes to Adaptation and Evolution in Natural Infection
Source: PLoS Pathog. 2014 Jul 31;10(7):e1004281. doi: 10.1371/journal.ppat.1004281 (PMC4117599; doi:10.1371/journal.ppat.1004281)

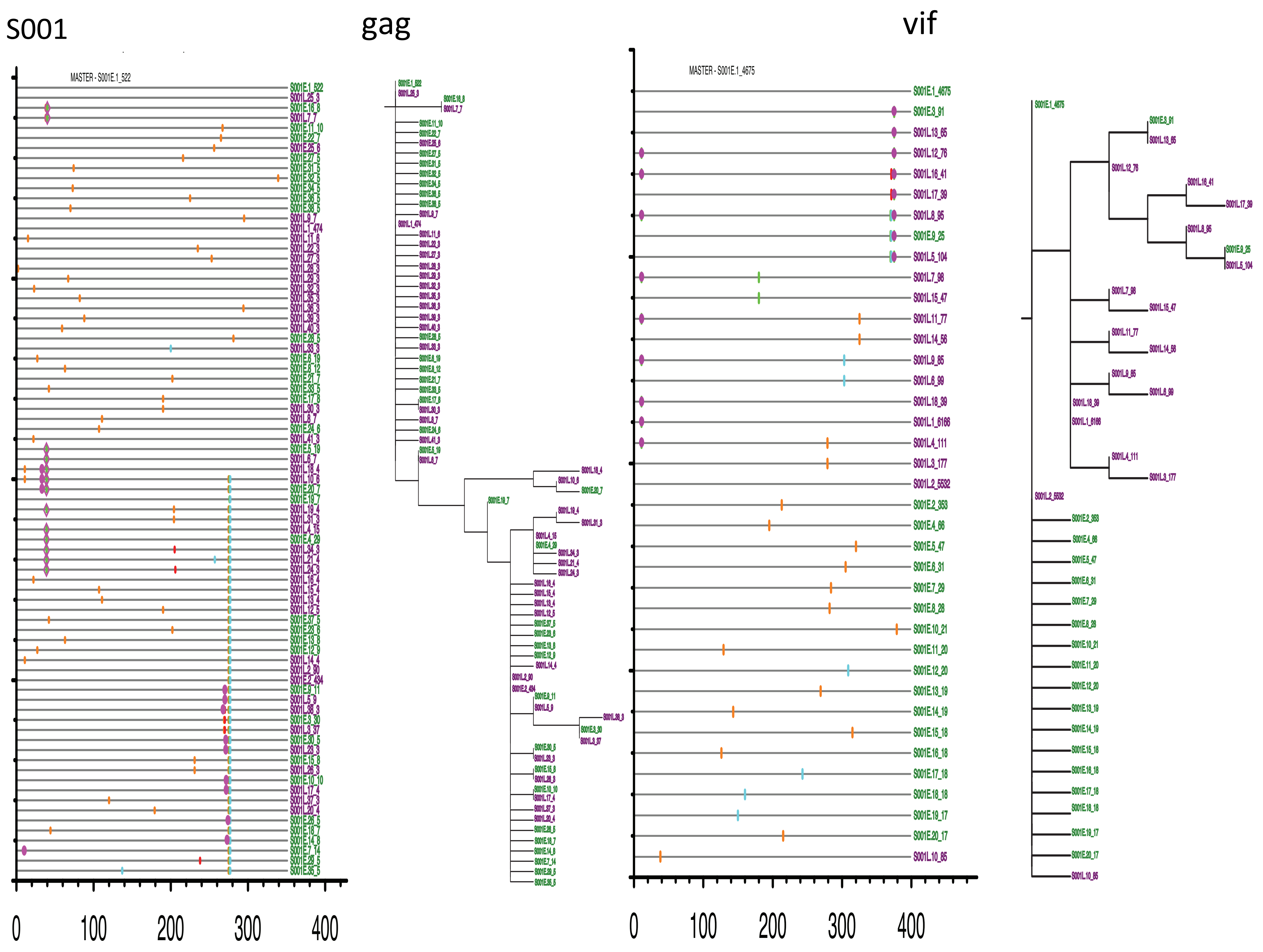
S001
gag
vif
MASTER - S001E.1_522
MASTER - S001E.1_4675
0
100
200
300
400

S002

gag

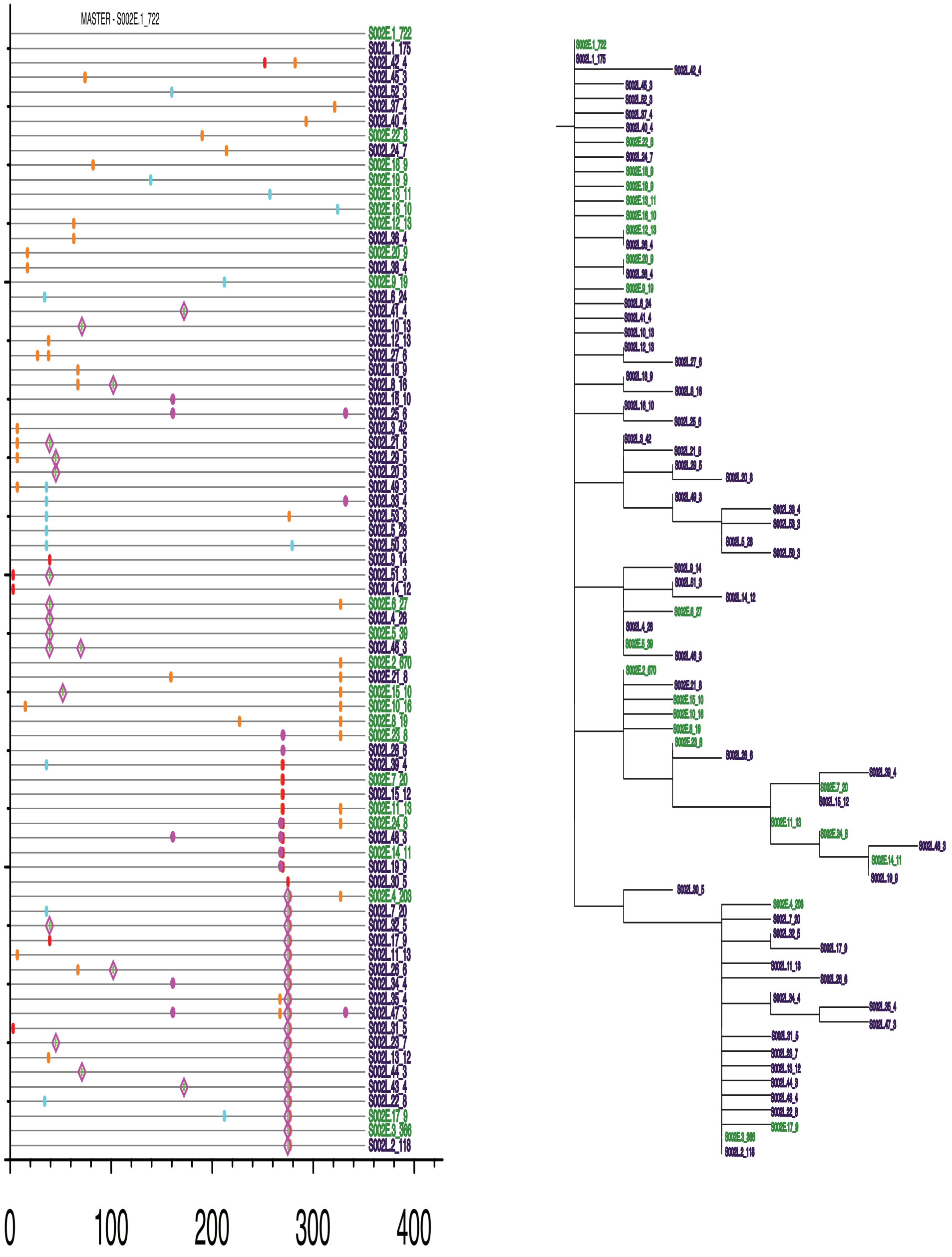
MASTER - S002E.1_722
0
100
200
300
400

vif

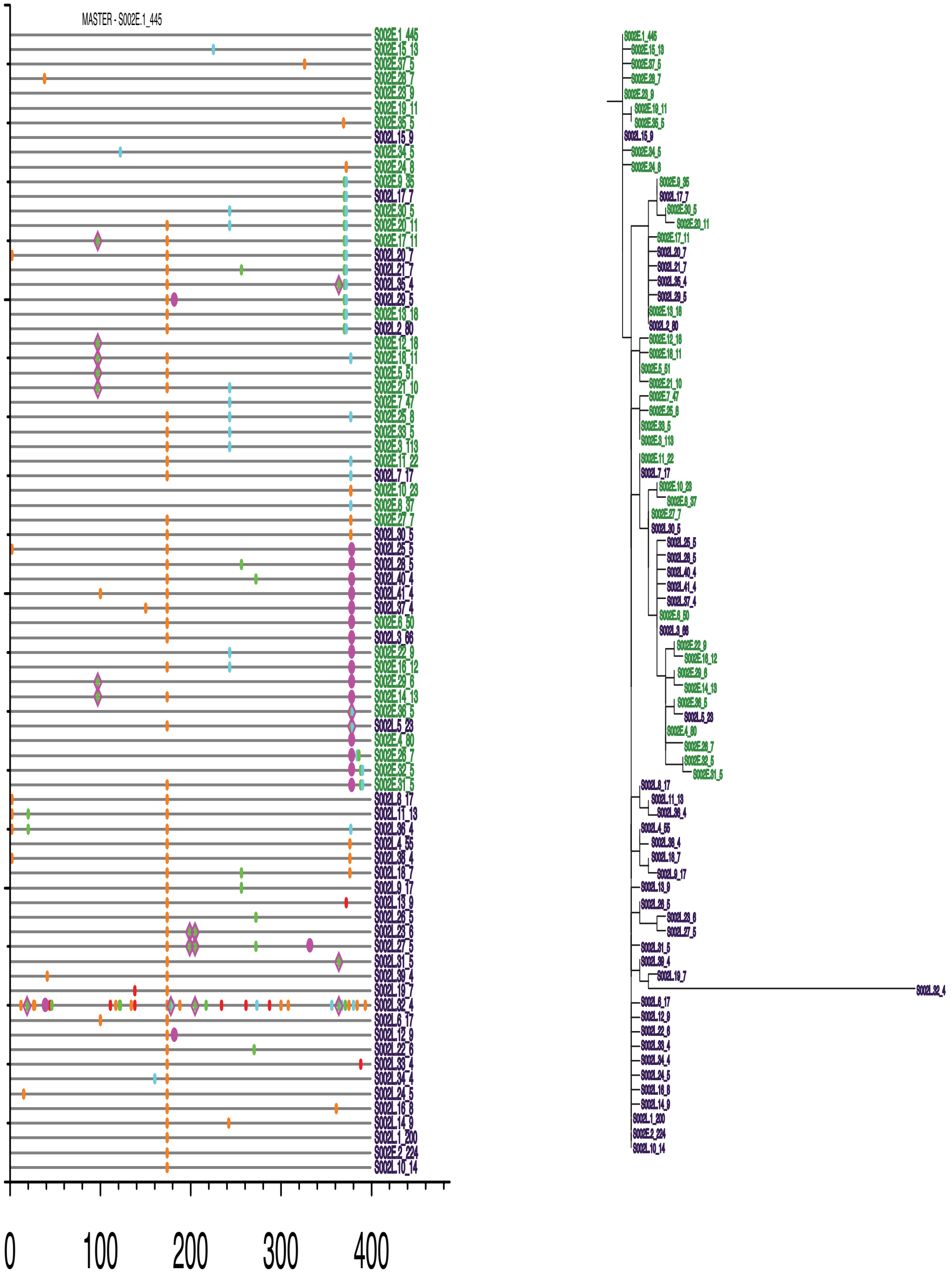
MASTER - S002E.1_445
0
100
200
300
400

S003

gag

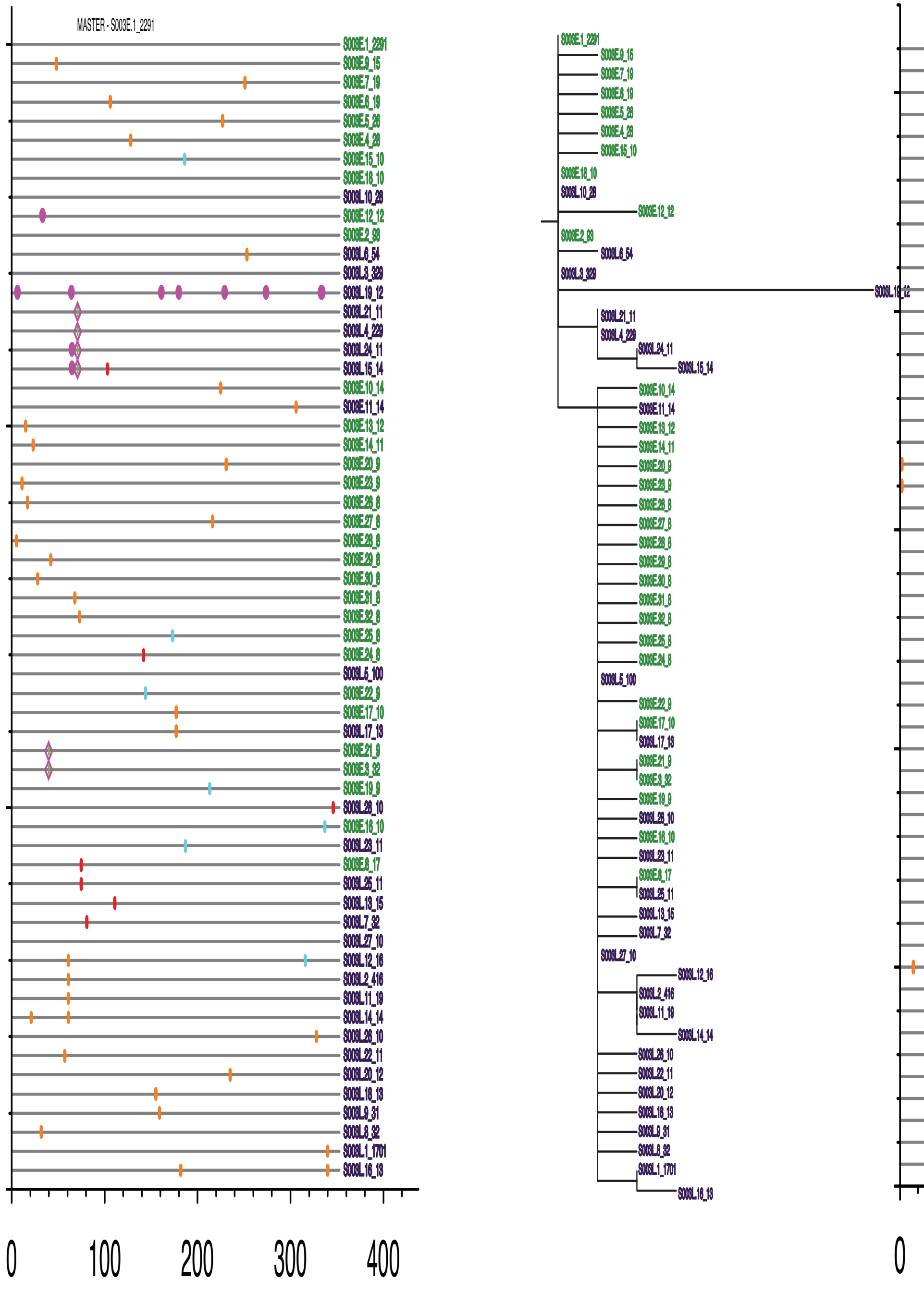

MASTER - S003E.1_2291
0
100
200
300
400

vif

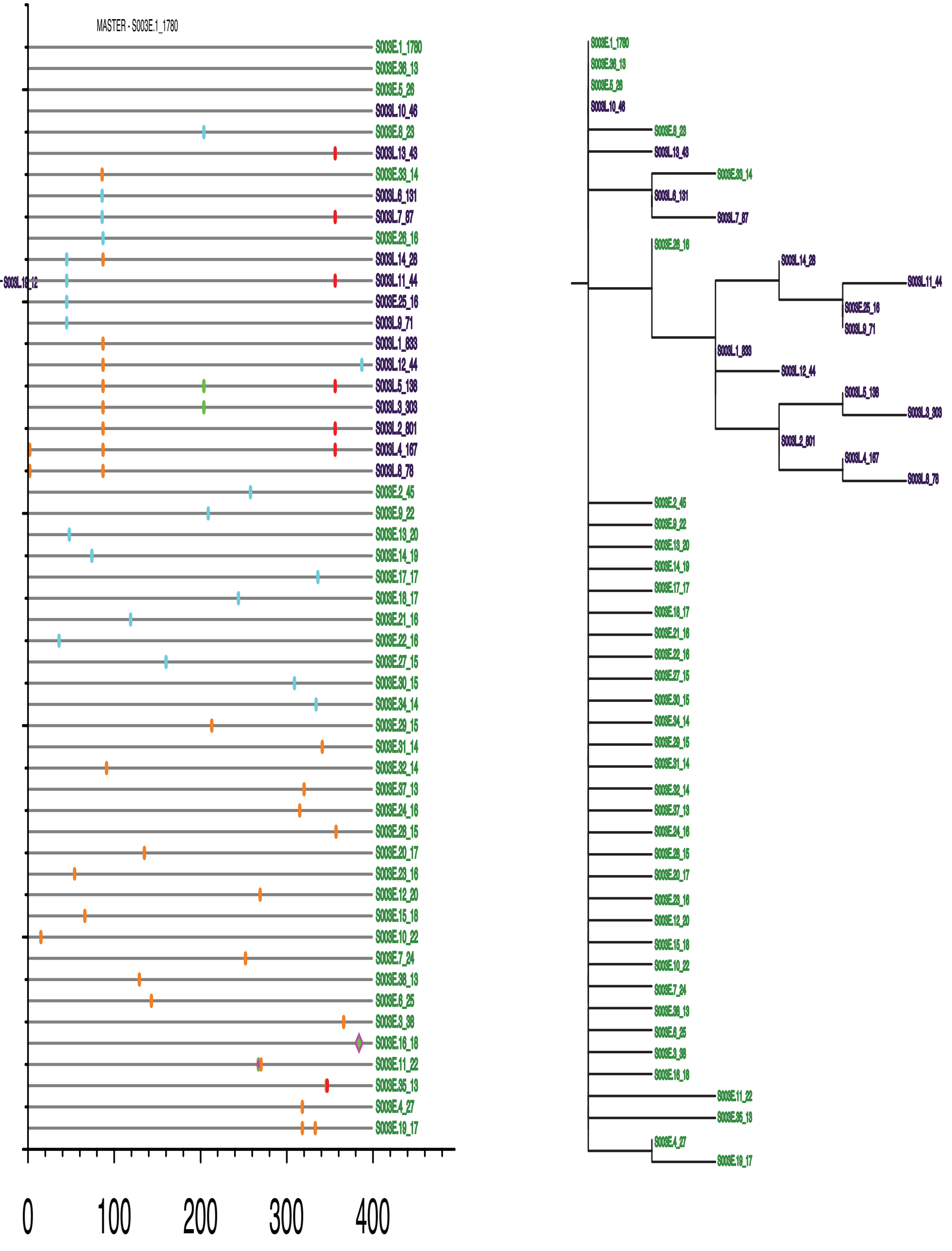

MASTER - S003E.1_1780
0
100
200
300
400

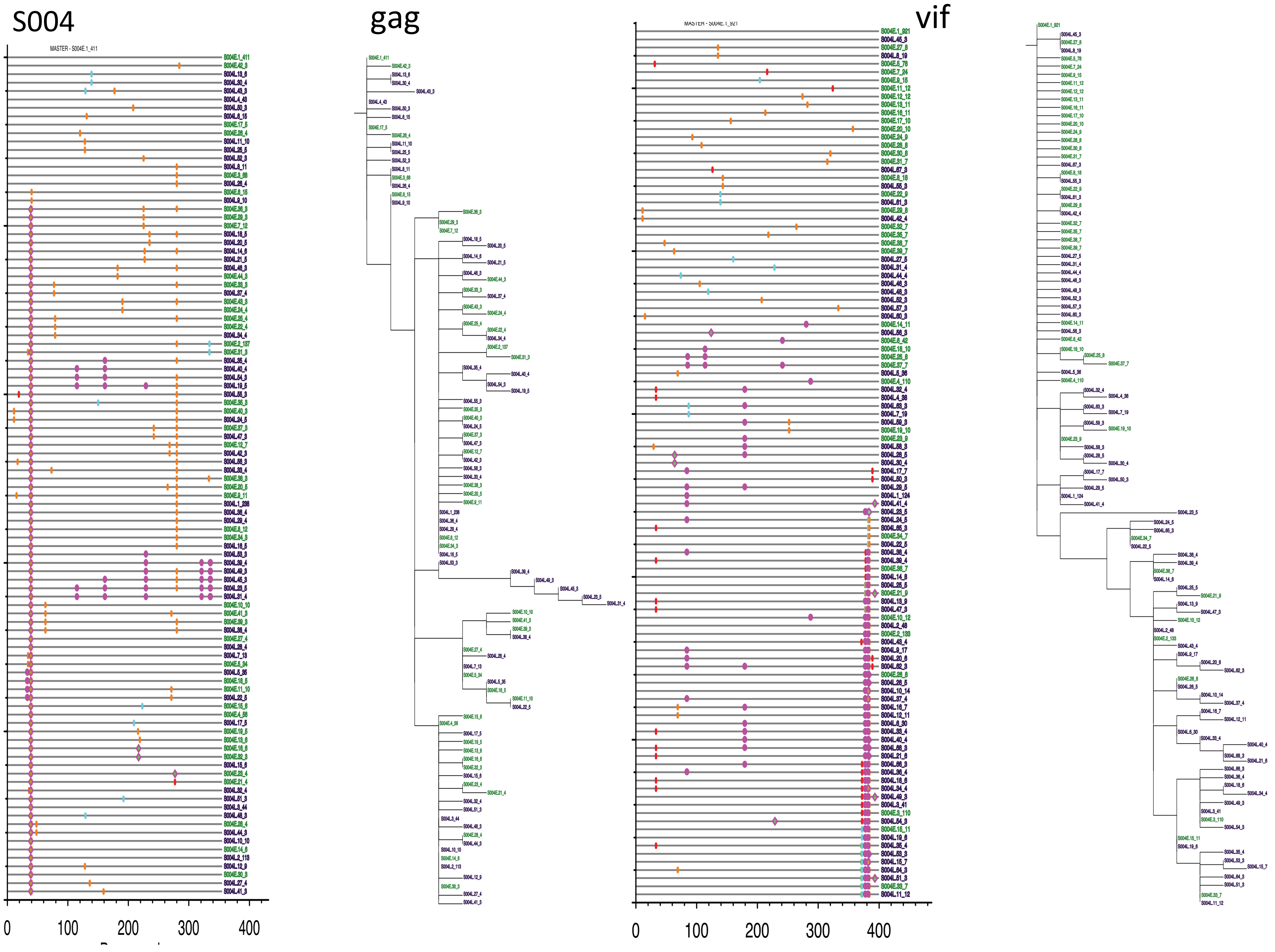
S004
gag
vif
0
100
200
300
400

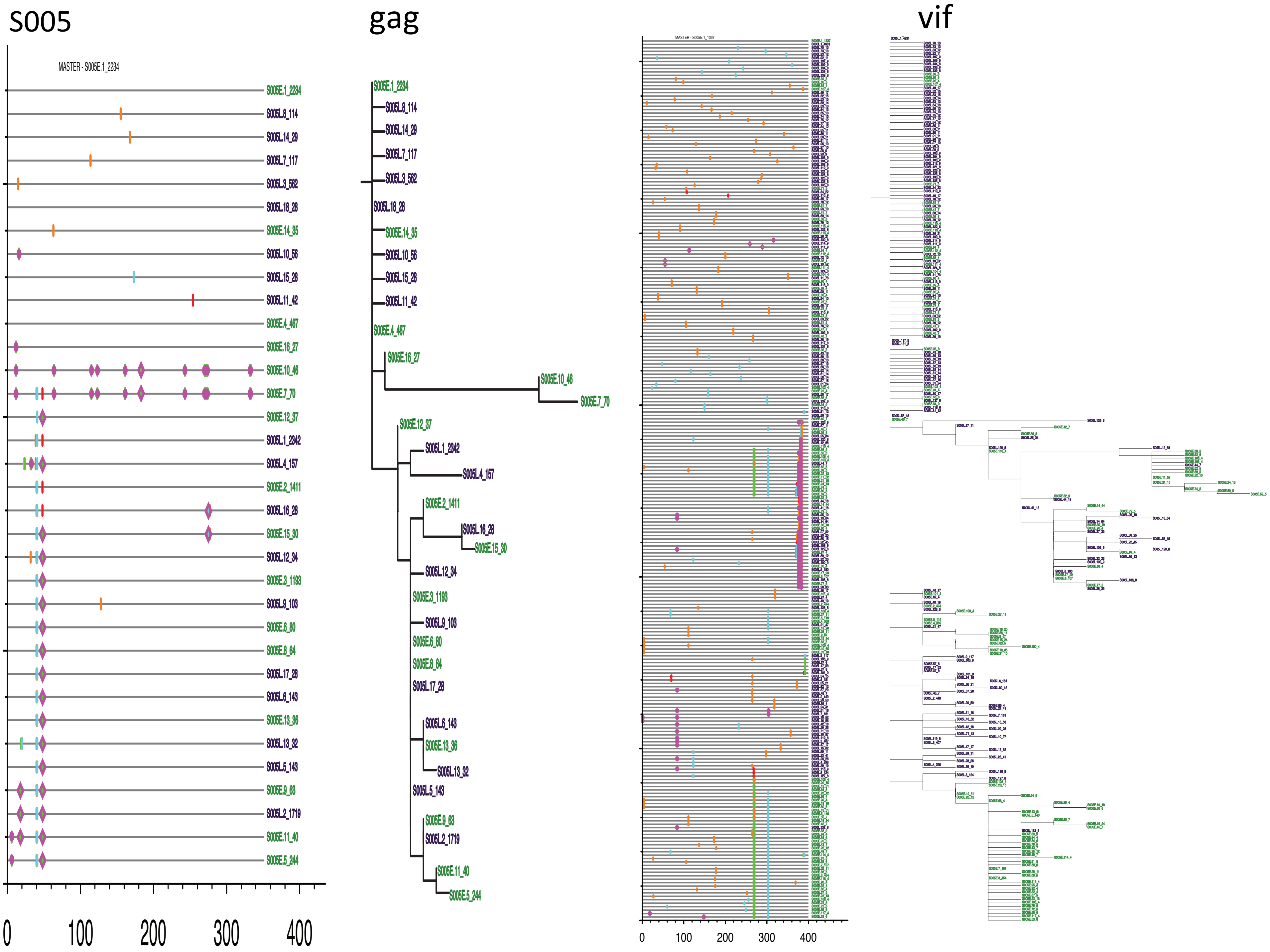
S005
gag
vif
MASTER - S005E.1_2234
S005E.1_2234
S005L8_114
S005L14_29
S005L7_117
S005L3_562
S005L18_28
S005E.14_35
S005L10_56
S005L15_28
S005L11_42
S005E.4_467
S005E.16_27
S005E.10_46
S005E.7_70
S005E.12_37
S005L1_2342
S005L4_157
S005E.2_1411
S005L16_28
S005E.15_30
S005L12_34
S005E.3_1193
S005L9_103
S005E.6_80
S005E.8_64
S005L17_28
S005L6_143
S005E.13_36
S005L13_32
S005L5_143
S005E.9_63
S005L2_1719
S005E.11_40
S005E.5_244
0
100
200
300
400

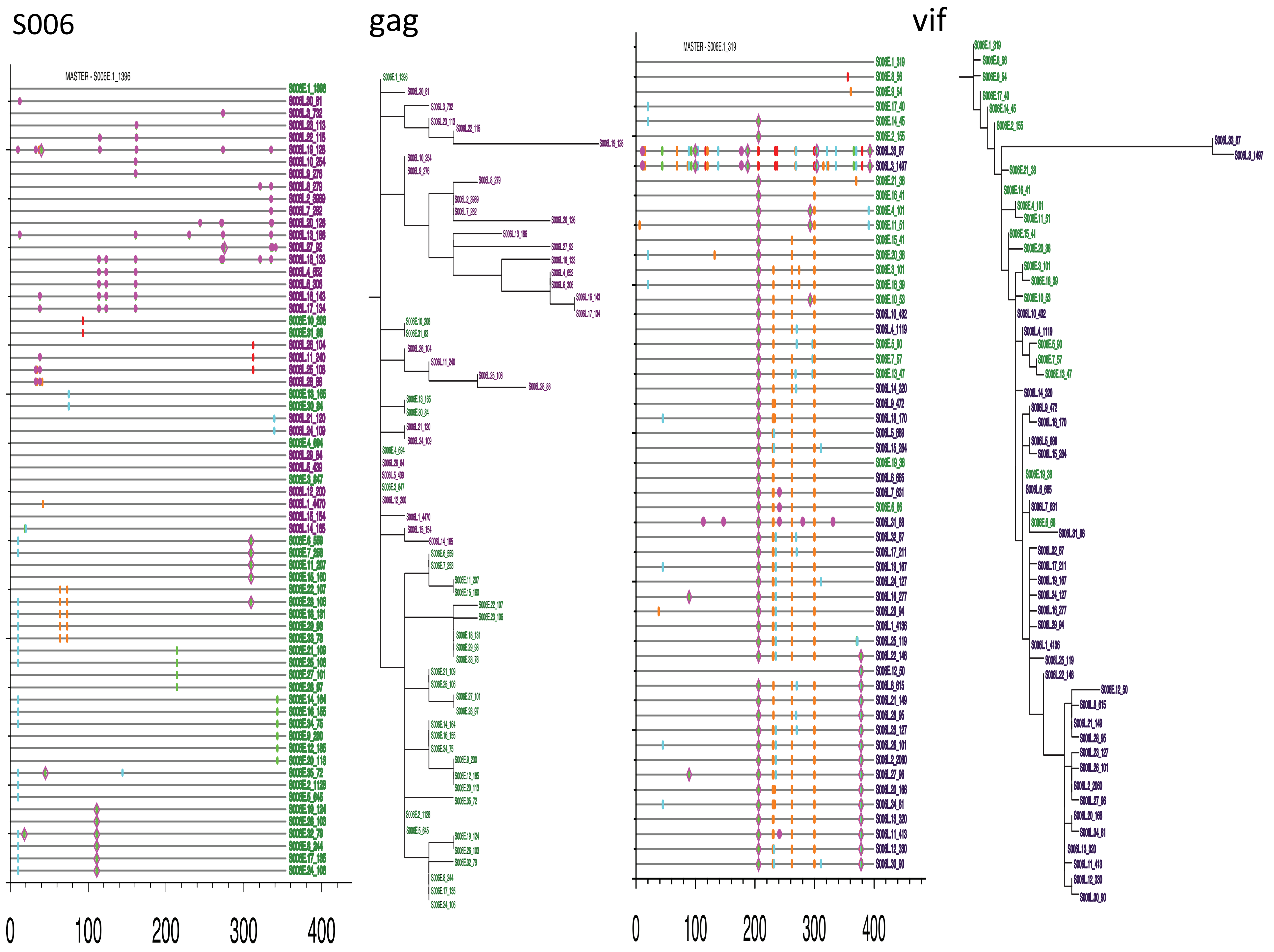
S006
gag
vif
MASTER - S006E.1_1396
MASTER - S006E.1_319
0
100
200
300
400

S007 gag vif

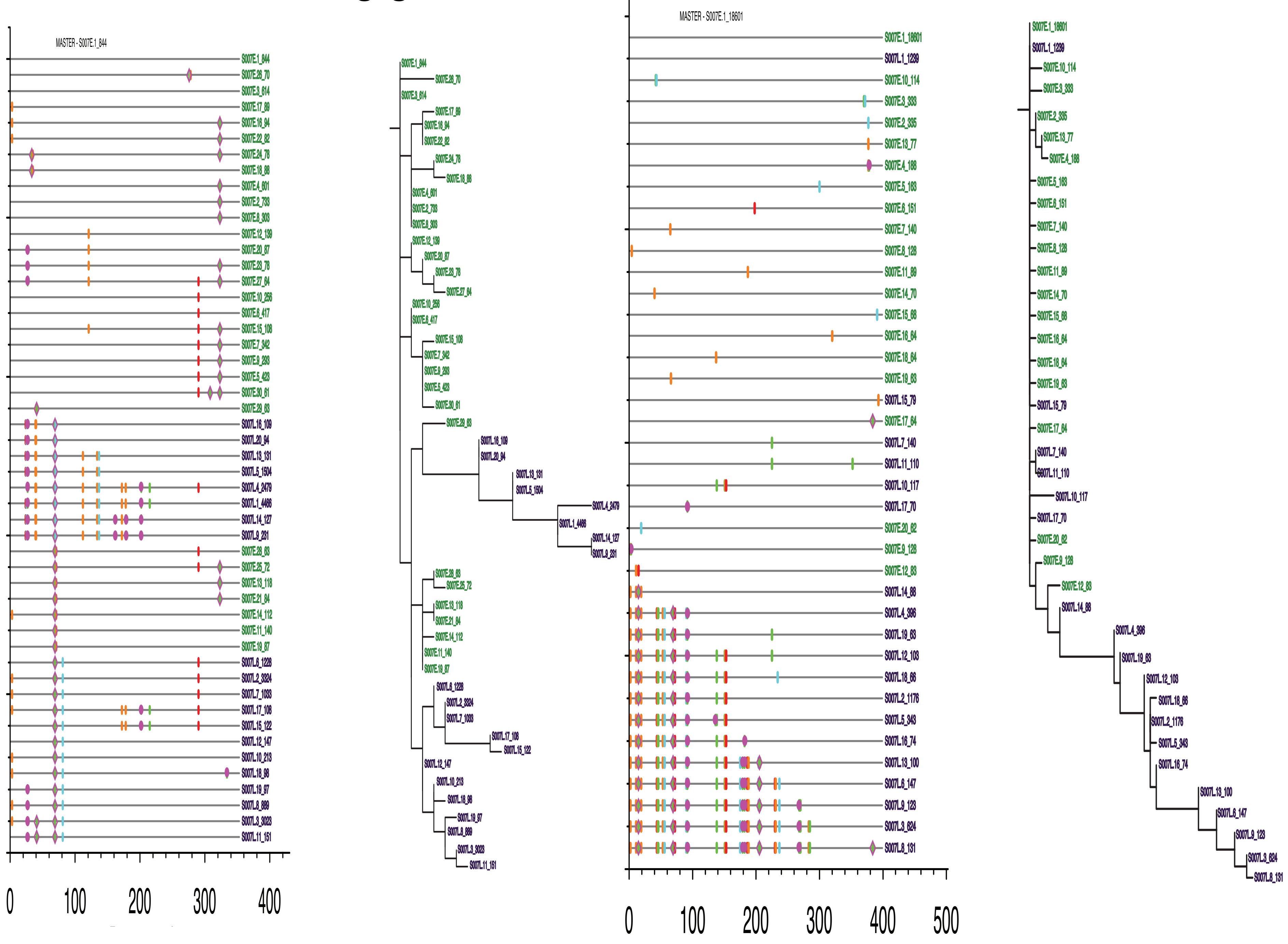

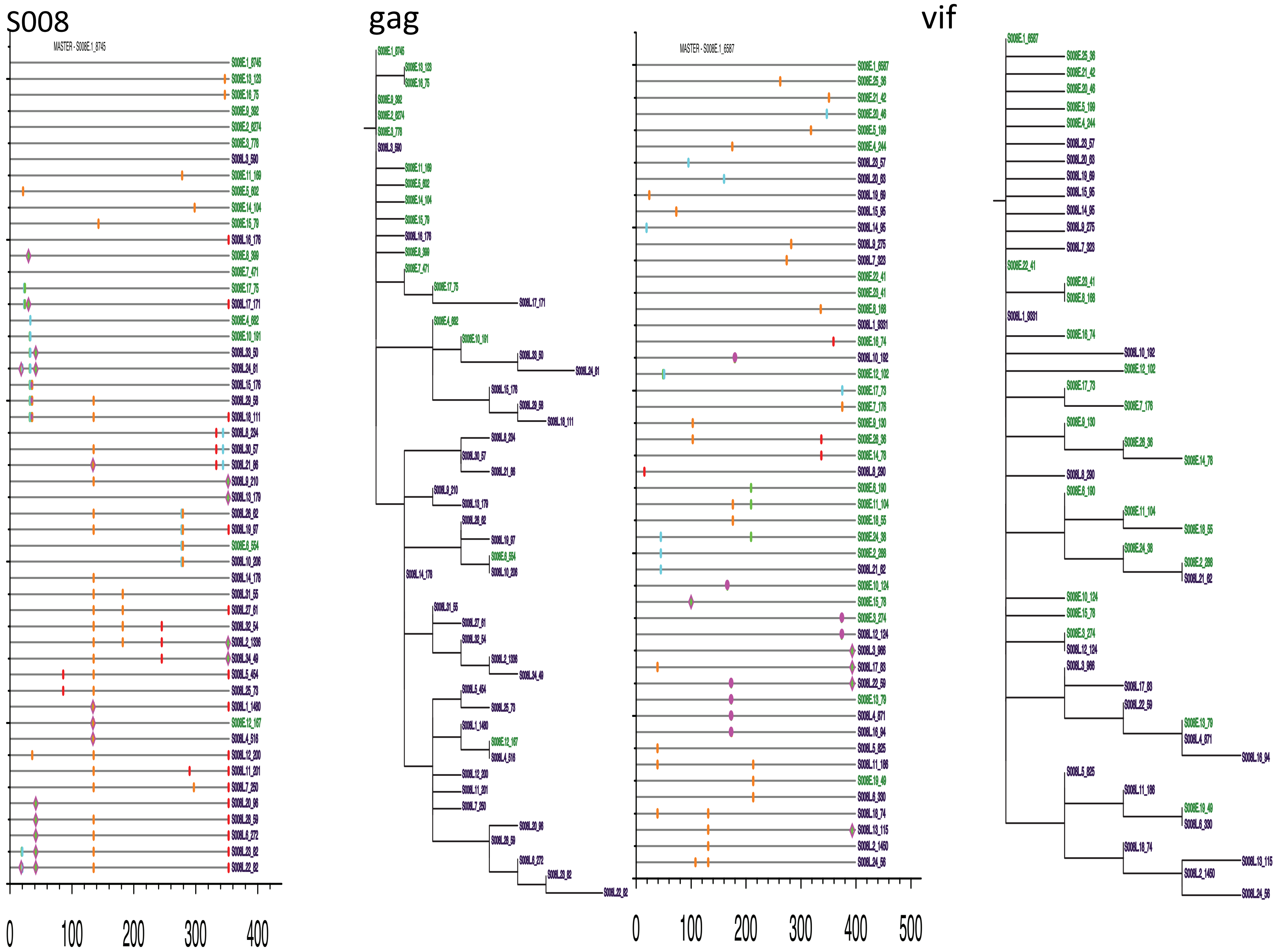

S008
gag
vif
MASTER - S008E.1_8745
MASTER - S008E.1_6587
0
100
200
300
400
500

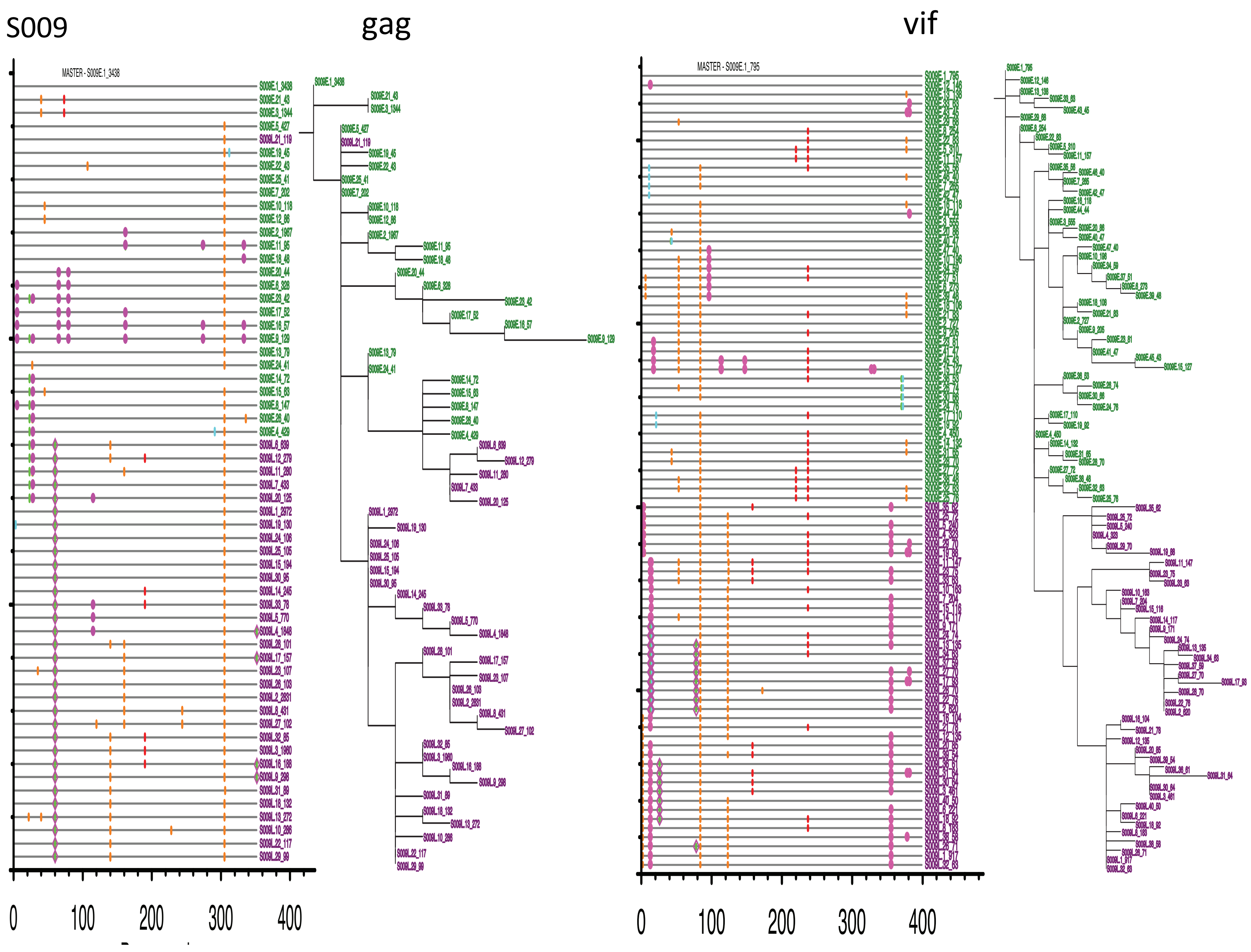
S009
gag
vif
MASTER - S009E.1_3438
MASTER - S009E.1_795
0
100
200
300
400

S010 gag vif

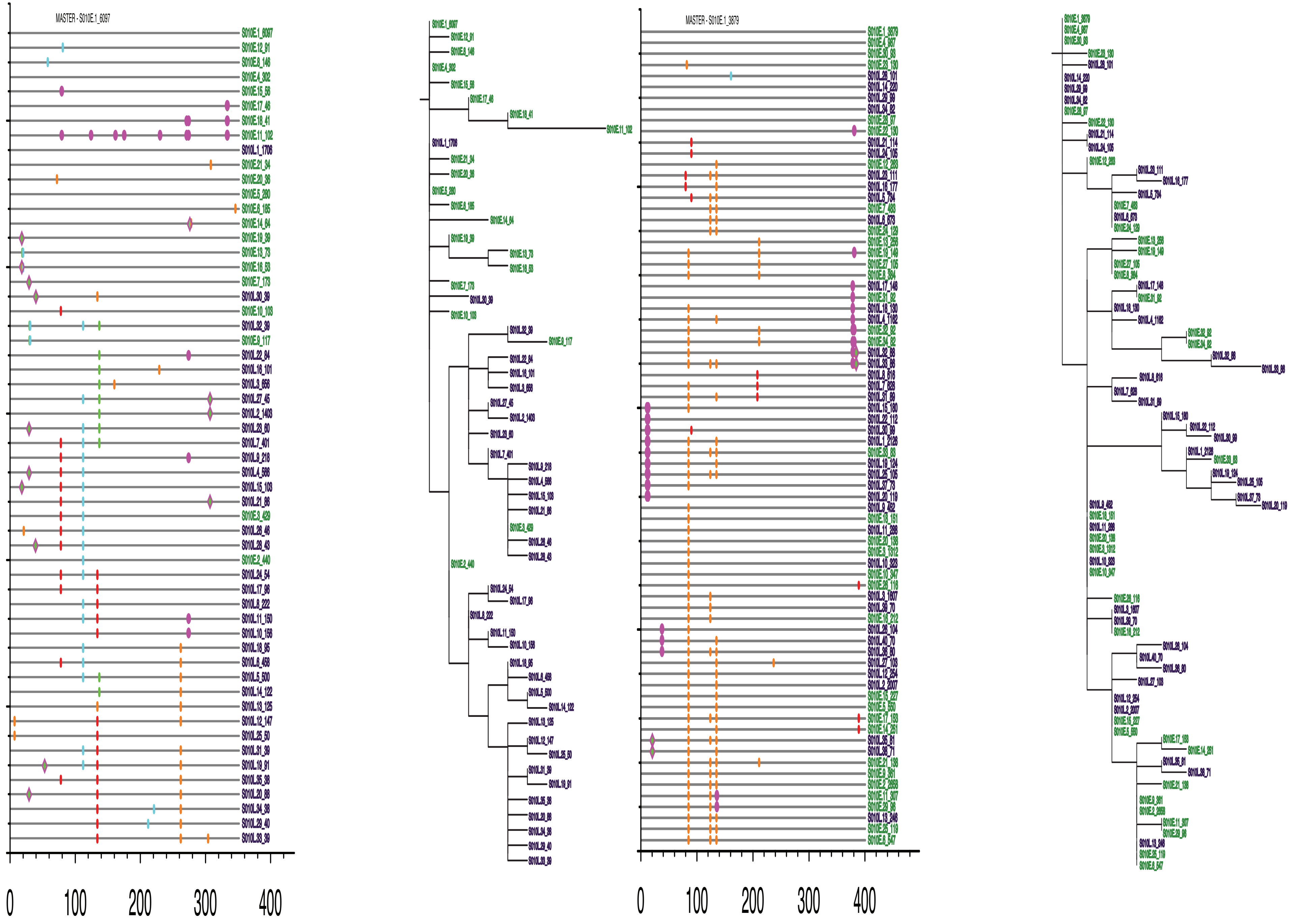

Supplement: Figure S3 — Neighbor joining phylogenetic trees and Highlighter plots of viral sequences from patients. Shown are Highlighter plots and neighbor-joining trees for the Gag and Vif genes of HIV-1 for all patients. Shown are the first (Day 0, green letters) and second (25 weeks, purple letters) time points during infection. The numbers present the number of collapsed sequences. The APOBEC3-mediated mutations are highlighted with lavender dots. Most of the evolved variants sampled at the later time points carry G-to-A mutations in the APOBEC3 trinucleotide context of the edited sites. The nucleotides that do not match with the master are assigned a color as given (A: Green; T: Red; G: Orange; C: Light blue; IUPAC codes (as regular characters): Dark blue; Gaps: Gray; Circle: APOBEC3, Diamond: G-to-A). (PDF) [file ppat.1004281.s003.pdf]
