# Supplementary material for: Human APOBEC3 Induced Mutation of Human Immunodeficiency Virus Type-1 Contributes to Adaptation and Evolution in Natural Infection
Source: PLoS Pathog. 2014 Jul 31;10(7):e1004281. doi: 10.1371/journal.ppat.1004281 (PMC4117599; doi:10.1371/journal.ppat.1004281)
Supplement: Table S1 — Patients' characteristics and estimated date of infection based on the onset of acute retroviral symptoms. (DOCX) [file ppat.1004281.s004.docx]

**Table S1.** Patients’ characteristics and estimated date of infection based on the onset of acute retroviral symptoms

| Subject | DAY_0 | Sex | RACE | SEX_W/MEN | 1STRNA (copy/ml) | 1ST CD4 (cells/ml) | CD4% | EDI* | EDI to Day 0 |
| --- | --- | --- | --- | --- | --- | --- | --- | --- | --- |
| S001 | 10/31/00 | Male | White | Yes | 1.76E+05 | 578 | 23 | 10/2/00 | 29 |
| S002 | 12/21/00 | Male | White | No | 2.87E+05 | 311 | 29 | 10/26/00 | 56 |
| S003 | 3/4/02 | Male | White | Yes | 5.13E+03 | 546 | 30 | 1/3/02 | 60 |
| S004 | 10/21/02 | Male | White | Yes | 5.76E+07 | 431 | 30 | 10/10/02 | 11 |
| S005 | 11/8/05 | Male | White | Yes | 1.70E+05 | 614 | 21 | 8/30/05 | 70 |
| S006 | 5/13/03 | Male | White | No | 4.46E+05 | 460 | 22 | 3/4/03 | 70 |
| S007 | 11/14/03 | Male | White | Yes | 7.79E+02 | 577 | 44 | 9/5/03 | 70 |
| S008 | 12/3/03 | Male | Black | Yes | 2.40E+05 | 650 | 21 | 10/31/03 | 33 |
| S009 | 4/6/04 | Male | White | Yes | 1.02E+05 | 502 | 37 | 1/27/04 | 70 |
| S010 | 7/2/04 | Male | White | Yes | 5.04E+05 | 760 | 25 | 4/23/04 | 70 |
| *EDI is an estimated date of infection | | | | | | | | | |
