# Supplementary material for: Human APOBEC3 Induced Mutation of Human Immunodeficiency Virus Type-1 Contributes to Adaptation and Evolution in Natural Infection
Source: PLoS Pathog. 2014 Jul 31;10(7):e1004281. doi: 10.1371/journal.ppat.1004281 (PMC4117599; doi:10.1371/journal.ppat.1004281)
Supplement: Table S2 — High-resolution HLA genotypes for the patients studied. (DOCX) [file ppat.1004281.s005.docx]

| **Table S2.**  High resolution HLA typing of the patients studied. | | | | | | | | |
| --- | --- | --- | --- | --- | --- | --- | --- | --- |
|  | **HLA-A** | **HLA-B** | **HLA-C** | **DPB1** | **DQA1** | **DQB1** | **DRB1** | **DRB4** |
| **S001** | A*02:01:01:01 | B*39:01:01:01 | C*06:02:01:01 | DPB1*02:01:02 | DQA1*01:01:01G | DQB1*03:03:02:01 | DRB1*01:01:01 | DRB4*01:01:01G |
|  | A*03:01:01:01 | B*57:01:01 | C*07:02:01:01 | DPB1*03:01:01 | DQA1*02:01 | DQB1*05:01:01:01 | DRB1*07:01:01:01 | DRB4*01:01:01G |
| **S002** | A*01:01:01:01 | B*07:02:01 | C*07:01:01 | DPB1*02:01:02 | DQA1*01:02:01:01 | DQB1*06:02:01 | DRB1*15:01:01:01 | DRB5*01:01:01 |
|  | A*24:02:01:01 | B*08:01:01 | C*07:02:01:01 | DPB1*04:01:01:01 | DQA1*01:02:01:01 | DQB1*06:02:01 | DRB1*15:01:01:01 | DRB5*01:01:01 |
| **S003** | A*03:01:01:01 | B*13:02:01 | C*04:01:01:01 | DPB1*04:01:01:01 | DQA1*01:02:01:01 | DQB1*03:01:01:01 | DRB1*01:03 | DRB5*01:01:01 |
|  | A*03:01:01:01 | B*35:01:01:01 | C*06:02:01:01 | DPB1*04:01:01:01 | DQA1*05:01:01G | DQB1*06:02:01 | DRB1*15:01:01:01 | DRB5*01:01:01 |
| **S004** | A*32:01:01 | B*40:01:01 | C*03:04:01:01 | DPB1*04:01:01:01 | DQA1*01:02:01:01 | DQB1*03:01:01:01 | DRB1*11:01:01 | DRB3*02:02:01:01 |
|  | A*68:01:02 | B*44:02:01:01 | C*07:01:01 | DPB1*04:01:01:01 | DQA1*05:01:01G | DQB1*06:02:01 | DRB1*15:01:01:01 | DRB5*01:01:01 |
| **S005** | A*02:01:01:01 | B*07:02:01 | C*02:02:02 | DPB1*04:01:01:01 | DQA1*01:01:01G | DQB1*03:02:01 | DRB1*01:02:01 | DRB4*01:01:01G |
|  | A*03:01:01:01 | B*14:02:01 | C*07:02:01:01 | DPB1*06:01 | DQA1*03:01:01G | DQB1*05:01:01:01 | DRB1*04:04:01 | DRB4*01:01:01G |
| **S006** | A*11:01:01 | B*39:05:01 | C*05:01:01:01 | DPB1*01:01:01 | DQA1*01:02:01:01 | DQB1*02:02 | DRB1*04:05:01 | DRB3*03:01:01 |
|  | A*68:03:01 | B*44:02:01:01 | C*07:02:01:01 | DPB1*04:01:01:01 | DQA1*03:01:01G | DQB1*05:01:01:01 | DRB1*13:02:01 | DRB4*01:01:01G |
| **S007** | A*03:01:01:01 | B*40:02:01 | C*02:02:02 | DPB1*04:02:01:01 | DQA1*02:01 | DQB1*02:02 | DRB1*07:01:01:01 | DRB3*02:02:01:01 |
|  | A*29:02:01:01 | B*44:03:01 | C*16:01:01 | DPB1*13:01 | DQA1*05:01:01G | DQB1*03:01:01:01 | DRB1*13:05:01 | DRB4*01:01:01G |
| **S008** | A*02:01:01:01 | B*44:02:01:01 | C*05:01:01:01 | DPB1*03:01:01 | DQA1*03:01:01G | DQB1*03:02:01 | DRB1*04:04:01 | DRB4*01:01:01G |
|  | A*23:01:01 | B*50:02 | C*06:02:01:01 | DPB1*11:01:01 | DQA1*03:01:01G | DQB1*04:02:01 | DRB1*04:06:01 | DRB4*01:01:01G |
| **S009** | A*02:01:01:01 | B*07:02:01 | C*06:02:01:01 | DPB1*01:01:01 | DQA1*01:01:01G | DQB1*03:01:01:01 | DRB1*01:01:01 | DRB4*01:01:01G |
|  | A*02:05:01 | B*57:01:01 | C*07:02:01:01 | DPB1*04:02:01:01 | DQA1*03:01:01G | DQB1*05:01:01:01 | DRB1*04:08:01 | DRB4*01:01:01G |
| **S010** | A*01:01:01:01 | B*13:02:01 | C*06:02:01:01 | DPB1*10:01 | DQA1*02:01 | DQB1*02:02 | DRB1*07:01:01:01 | DRB4*01:01:01G |
|  | A*02:01:01:01 | B*57:01:01 | C*06:02:01:01 | DPB1*11:01:01 | DQA1*02:01 | DQB1*03:03:02:01 | DRB1*07:01:01:01 | DRB4*01:01:01G |
