# Supplementary material for: Human APOBEC3 Induced Mutation of Human Immunodeficiency Virus Type-1 Contributes to Adaptation and Evolution in Natural Infection
Source: PLoS Pathog. 2014 Jul 31;10(7):e1004281. doi: 10.1371/journal.ppat.1004281 (PMC4117599; doi:10.1371/journal.ppat.1004281)
Supplement: Table S3 — APOBEC3H haplotypes for the patients studied. (DOCX) [file ppat.1004281.s006.docx]

| **Table S3. APOBEC3H haplotypes for the patients studied** | | | | | | |
| --- | --- | --- | --- | --- | --- | --- |
|  |  |  |  |  |  |  |
| Patients | N15Δ | R18L | G105R | K121D/E | E178D | Haplotype |
| S001 | NN | RR | GG | KK | EE | I |
| S002 | ΔΔ | RL | GR | DD | ED | IV/VI |
| S003 | ΔN | RL | RR | DD | DD | II/IV |
| S004 | NN | RR | GR | DD | ED | I/II |
| S005 | NN | RR | GG | KK | EE | I |
| S006 | ΔΔ | RL | GR | KD | ED | I/IV |
| S007 | NN | RR | GG | KK | EE | I |
| S008 | ΔN | RR | RR | DD | DD | II/III |
| S009 | NN | RR | GG | KD | EE | I/I |
| S010 | NN | RR | GG | KK | EE | I |
