# Supplementary material for: Human APOBEC3 Induced Mutation of Human Immunodeficiency Virus Type-1 Contributes to Adaptation and Evolution in Natural Infection
Source: PLoS Pathog. 2014 Jul 31;10(7):e1004281. doi: 10.1371/journal.ppat.1004281 (PMC4117599; doi:10.1371/journal.ppat.1004281)
Supplement: Table S4 — Number of sequence reads in the process of error correction and reconstruction of haplotypes. After error correction, the reads were aligned and trimmed to encompass the greatest coverage, then collapsed into unique haplotypes. The median number of the minimal inferred candidate haplotypes present in the virus population. (DOCX) [file ppat.1004281.s007.docx]

| **Table S4.** Number of sequence reads in the process of error correction and reconstruction of haplotypes. | | | | | | | | | | | |
| --- | --- | --- | --- | --- | --- | --- | --- | --- | --- | --- | --- |
| **Original Alignment** | | | **Length Filtered Alignment** | | | **Collapsed Alignment** | | | **Collapsed seqs for 0.2% Alignment** | | |
| **Alignment** | **Num. Seqs reads** | | **Alignment** | **Num. Seqs filtered** | | **Alignment** | **Num. Seqs collapsed** | | **Alignment** | **Min. num. Seqs Collapsed** | |
|  | **gag** | **vif** |  | **gag** | **vif** |  | **gag** | **vif** |  | **gag** | **vif** |
| align_S001E.fasta | 44219 | 44999 | S001E.txt | 2505 | 8700 | S001E_Collapsed | 1005 | 1631 | S001E_Collapsed | 5 | 17 |
| align_S001L.fasta | 36866 | 51098 | S001L.txt | 1264 | 18510 | S001L_Collapsed | 482 | 2583 | S001L_Collapsed | 3 | 37 |
| align_S002E.fasta | 41035 | 45971 | S002E.txt | 4061 | 2603 | S002E_Collapsed | 1296 | 1132 | S002E_Collapsed | 8 | 5 |
| align_S002L.fasta | 46212 | 52324 | S002L.txt | 1489 | 1893 | S002L_Collapsed | 751 | 1143 | S002L_Collapsed | 3 | 4 |
| align_S003E.fasta | 59232 | 53522 | S003E.txt | 3945 | 4494 | S003E_Collapsed | 796 | 1273 | S003E_Collapsed | 8 | 9 |
| align_S003L.fasta | 44269 | 43565 | S003L.txt | 4933 | 5469 | S003L_Collapsed | 1287 | 1846 | S003L_Collapsed | 10 | 11 |
| align_S004E.fasta | 40985 | 58012 | S004E.txt | 1476 | 3337 | S004E_Collapsed | 744 | 1249 | S004E_Collapsed | 3 | 7 |
| align_S004L.fasta | 51120 | 55193 | S004L.txt | 1589 | 1532 | S004L_Collapsed | 678 | 893 | S004L_Collapsed | 3 | 3 |
| align_S005E.fasta | 30582 | 84587 | S005E.txt | 13736 | 6538 | S005E_Collapsed | 4734 | 2175 | S005E_Collapsed | 27 | 13 |
| align_S005L.fasta | 30082 | 65320 | S005L.txt | 13195 | 14035 | S005L_Collapsed | 4675 | 3773 | S005L_Collapsed | 26 | 28 |
| align_S006E.fasta | 40715 | 59550 | S006E.txt | 35018 | 17948 | S006E_Collapsed | 16945 | 13033 | S006E_Collapsed | 70 | 36 |
| align_S006L.fasta | 46674 | 56148 | S006L.txt | 40096 | 37488 | S006L_Collapsed | 15259 | 12249 | S006L_Collapsed | 80 | 75 |
| align_S007E.fasta | 36238 | 58094 | S007E.txt | 30652 | 30372 | S007E_Collapsed | 16435 | 18436 | S007E_Collapsed | 61 | 61 |
| align_S007L.fasta | 51815 | 59385 | S007L.txt | 46279 | 30248 | S007L_Collapsed | 14432 | 3788 | S007L_Collapsed | 93 | 60 |
| align_S008E.fasta | 37759 | 51873 | S008E.txt | 34298 | 28142 | S008E_Collapsed | 6864 | 6067 | S008E_Collapsed | 69 | 56 |
| align_S008L.fasta | 43007 | 52697 | S008L.txt | 24409 | 17324 | S008L_Collapsed | 10894 | 4495 | S008L_Collapsed | 49 | 35 |
| align_S009E.fasta | 37227 | 55366 | S009E.txt | 34947 | 24803 | S009E_Collapsed | 11082 | 12724 | S009E_Collapsed | 70 | 50 |
| align_S009L.fasta | 28006 | 32587 | S009L.txt | 19999 | 20061 | S009L_Collapsed | 6088 | 8844 | S009L_Collapsed | 40 | 40 |
| align_S010E.fasta | 36412 | 41649 | S010E.txt | 17027 | 38799 | S010E_Collapsed | 4499 | 14585 | S010E_Collapsed | 34 | 78 |
| align_S010L.fasta | 36407 | 37887 | S010L.txt | 18844 | 34180 | S010L_Collapsed | 7183 | 13046 | S010L_Collapsed | 38 | 68 |
