# Supplementary material for: Human APOBEC3 Induced Mutation of Human Immunodeficiency Virus Type-1 Contributes to Adaptation and Evolution in Natural Infection
Source: PLoS Pathog. 2014 Jul 31;10(7):e1004281. doi: 10.1371/journal.ppat.1004281 (PMC4117599; doi:10.1371/journal.ppat.1004281)
Supplement: Table S5 — Positively and negatively selected codon sites in the Gag and Vif genes of HIV-1. (DOCX) [file ppat.1004281.s008.docx]

**Table S5.** Positive and negative selected codon sites in HIV-1 Gag and Vif

| **Gag** |  |  |  |  |  |
| --- | --- | --- | --- | --- | --- |
| **Patient** | **Codon #** | **Triplet** | **APOBEC** | **Selection** | **Potential HLA epitope** |
| S001 | 114 | AAG | No | Negative |  |
| S002 | 102 | GAC | No | Negative |  |
|  | 210 | GCA | No | Negative |  |
| S004 | 113 | AAA | No | Positive | B*0801 |
| S006 | 204 | GAG |  | Negative |  |
| S008 | 204 | ACC | No | Negative |  |
|  | 218 | GCA | No | Positive | No |
| S009 | 164 | TTC | No | Negative |  |
|  | 218 | GTG |  | Negative |  |
| S010 | 107 | GAG | No | Negative |  |
|  | 138 | CTT | No | Negative |  |
|  | 192 | GGG | Yes | Negative |  |
|  | 203 | GAG | No | Negative |  |

| **Vif** |  | |  | |  | |  |  | |
| --- | --- | --- | --- | --- | --- | --- | --- | --- | --- |
| **Patient** | **Codon #** | **Triplet** | | **APOBEC** | | **Selection** | **Potential HLA epitope** | |  |
| S001 | 36 | AGG | | Yes | | Positive | No | |  |
|  | 165 | AGT | | No | | Negative |  | |  |
| S002 | 78 | GAC | | No | | Negative |  | |  |
|  | 90 | AGA | | No | | Negative |  | |  |
|  | 113 | GAT | | No | | Negative |  | |  |
| S004 | 43 | CAC | | No | | Negative |  | |  |
|  | 55 | GTA | | No | | Negative |  | |  |
|  | 60 | GGG | | Yes | | Negative |  | |  |
|  | 92 | AGG | | Yes | | Positive | A*6801 B*4402 | |  |
| S005 | 50 | AAA | | No | | Negative |  | |  |
|  | 60 | GGG | | Yes | | Negative |  | |  |
|  | 73 | CAT | | No | | Negative |  | |  |
|  | 133 | TGT | | No | | Negative |  | |  |
|  | 156 | CCA | | No | | Negative |  | |  |
| S006 | 47 | ACT | | No | | Negative |  | |  |
| S008 | 45 | GAA | | No | | Negative |  | |  |
|  | 102 | CTA | | No | | Positive | A*0201 B*4402 | |  |
| S009 | 37 | GGA | | Yes | | Positive | No | |  |
|  | 50 | AAA | | No | | Positive | A*0205 | |  |
|  | 60 | GAA | | No | | Negative |  | |  |
|  | 111 | TAC | | No | | Negative |  | |  |
|  | 151 | GAA | | Yes | | Positive | A*0205 | |  |
| S010 | 60 | GGA | | No | | Negative |  | |  |
